# Review of wing morphology in fossil and modern species of humpbacked flies (Diptera: Phoridae)

**DOI:** 10.1186/s12915-025-02376-8

**Published:** 2025-10-07

**Authors:** Mélanie C. M. Herbert, André Nel, Brian V. Brown, Antonio Arillo, Brendon E. Boudinot, Mónica M. Solórzano-Kraemer

**Affiliations:** 1 1Paläontologie und Historische Geologie, Senckenberg Forschunginstitut und Naturmuseum, Senckenberganlage 25, Frankfurt-Am-Main, 60325 Germany; 2 Institut Systématique Evolution Biodiversité (ISYEB), Muséum National d’Histoire Naturelle, CNRS, Sorbonne Université, EPHE, Université Des Antilles, Paris, France; 3 Department of Entomology, Natural History Museum of Los Angeles County, 900 Exposition Blvd, Los Angeles, CA 90007 USA; 4 Departamento de Biodiversidad, Facultad de Biología, Universidad Complutense, Ecología y Evolucíon, Madrid, Spain; 5 Entomology II, Abteilung Terrestrische Zoologie, Senckenberg Forschungsinstitut und Naturmuseum, Senckenberganlage 25, Frankfurt-Am-Main, 60325 Germany

**Keywords:** Cretaceous, Wing vein terminology, New species, Amber, Brachycera, Insecta

## Abstract

**Background:**

The wing veins of known fossil and living phorids are reduced, making them difficult to homologise. Consequently, different interpretations have led to much confusion over the years. However, veins are crucial for phylogenetic and taxonomy studies, especially for fossils. We addressed these challenges by studying Cretaceous specimens, which exhibit fewer reductions in wing veins compared to modern fauna, along with post-Cretaceous specimens that display recent wing patterns. Furthermore, we examined related families such as Ironomyiidae, Platypezidae, Opetiidae, and Lonchopteridae to show wing similarities to the Phoridae.

**Results:**

We propose two wing models that include the majority of veins found in each taxon. The *early model* (Phoridae *sensu lato*, including Sciadocerinae + “†Prioriphorinae”) consists of most Cretaceous species, except †*Metopina goeleti* from New Jersey amber, which displays a recent pattern. The *recent model* (Phoridae *sensu stricto*, i.e. Euphorida) is present in the majority of recent phorids. Based on this new interpretation, we re-evaluate three holotypes of Phoridae: †*Euliphora grimaldii*, †*Prioriphora schroederhohenwarthi*, and †*Ulrichophora lobata*. Additionally, we described a Phoridae specimen belonging to †*Prioriphora* in the Fouras-Bois Vert amber (France) and a new genus within the Ironomyiidae family in the San Just amber (Spain).

**Conclusion:**

The newly proposed wing models facilitate rapid identification of Phoridae wing veins based on their degree of reduction. The two newly described specimens are the first records of these taxa in their respective localities.

**Supplementary Information:**

The online version contains supplementary material available at 10.1186/s12915-025-02376-8.

## Background

Species of the family Phoridae Curtis, including the subfamily Sciadocerinae Schmitz [1–4], are commonly known as “humpbacked flies” or “scuttle flies”. Phorids are small cyclorrhaphan flies, between 0.4 and 6 mm, with an astonishing diversity. Although far from being completely known, they already contain 4464 extant described species [5], but the estimate is much higher, probably between 40 and 50,000 species [6]. Conversely, the fossil record is much less rich. 

Wing venation plays an essential role in the identification and study of cyclorrhaphan families, especially in the study of fossil remains due to the abundance of wings which, besides showing distinct characters, are easy to describe [7–16]. However, the history of vein terminology is extensive and has been the subject of considerable debate. The first comprehensive model for dipteran wing structures was established by Comstock and Needham [17]. The groundplan for Diptera was comprehensively revised by McAlpine et al. [8] in terms of the medial and cubital veins, and several changes were made afterwards (i.e. [18–20]). The last standard terminology was created by Cumming and Wood [13], which was built upon an unpublished handout by Saigusa [21] distributed at the 6^th^ International Congress of Dipterology. In recent years, new research employing microtomographic x-ray scanning (µ-CT) has clarified the identity of the main postcubital vein (PCu vein) [22] and an intercalary longitudinal vein (fiv vein) in all Neoptera and especially Diptera, resulting in yet further refinements to wing venations terminology [16].

Adult Phoridae are easily identifiable by their modified wing venation, primarily due to the reduction of their radial and costal veins. The characters used for the classification of fossil specimens within the family are essentially based on the wing morphological structures [23–25]. Thus, despite vein reduction, wings are essential structures to hypothesise phylogenetic relationships between living and fossil phorids and their outgroups Platypezidae Fallén, Ironomyiidae McAlpine and Martin, Opetiidae Rondani, and Lonchopteridae Macquart [25].

Establishing a terminology for homologous wing veins, not only for the fossil phorids but also for the living ones, is a special challenge due to their highly derived wing venation, which is the result of a significant reduction of wing venation compared to other families of Diptera. The preferred labelling system for venation has been debated for many years, revealing inconsistencies across various authors [25–30]. The difficulties arise from two main problems. The first involves applying nomenclature derived from the wing venation ground plan of Diptera to Phoridae [8, 11, 13, 17–21], resulting in multiple interpretations of wing terminology (Table 1), particularly concerning the posterior part of the wing [1, 3, 23, 26, 28, 31]. The second involves reduced wing venation, especially the vanishing of the proximal parts of the medial, cubital, and anal veins, resulting in the absence of connections. These problems, coupled with considerable variability, especially in extant genera, present a challenge for the consistent labelling of phorid veins and establishing a terminology reflecting wing venation homologies.

**Table 1:** Wing vein terminology is used for Diptera and within Diptera specific for Phoridae. In italic: Diptera wing vein terminology also used for Phoridae. Not italicised: Phoridae wing vein terminology used in the description of species from various authors. In bold black: the new combination terminology model. X: it is not considered as a vein; ?: vein no mentioned; /: vein not present

*Comstock and Needham (1898)*	*C*	*Sc*	*R*_*1*_	*R*_*S*_	*R*_*2*+*3*_	*R*_*4*+*5*_	*M*_*1*_	*M*_*2*_	*M*_*3*_	*M*_*4*_	*CuA*	*CuP*	*A*_*1*_	*A*_*2*_	*A*_*3*_
*McAlpine *et al*. (1981)*	*C*	*Sc*	*R*_*1*_	*R*_*S*_	*R*_*2*+*3*_	*R*_*4*+*5*_	*M*_*1*_	*M*_*2*_	*M*_*3*_	*CuA*_*1*_	*CuA*_*2*_	*CuP*	*A*_*1*_	*A*_*1*_	*A*_*2*_
*Wootton and Ennos (1989)*	*C*	*Sc*	*R*	*Rs*	*Rs*_*1*+*2*_	*Rs*_*3*+*4*_	*M*_*1*_	*M*_*2*_	*M*_*3*_	*M*_*4*_	*CuA*	*CuP*	*A*_*1*_	*A*_*2*_	*A*_*3*_
*Saigusa (2006)*	*C*	*Sc*	*R*_*1*_	*Rs*	*R*_*2*+*3*_	*R*_*4*+*5*_	*M*_*1*_	*M*_*2*_	*M*_*3*_	*M*_*4*_	*CuA*	*X*	*CuP*	*A*_*1*_	*A*_*2*_
*Chapman (1998, 2013)*	*C*	*Sc*	*R*_*1*_	*Rs*	*Rs*_*1*+*2*_	*Rs*_*3*+*4*_	*MA*_*1*_	*MA*_*2*_	*MP*_*1*_	*MP*_*2*_	*CuA*	*CuP*	*A*_*1*_	*A*_*2*_	*A*_*3*_
*Cumming and Wood (2017)*	*C*	*Sc*	*R*_*1*_	*R*_*S*_	*R*_*2*+*3*_	*R*_*4*+*5*_	*M*_*1*_	*M*_*2*_	*M*_*3*_	*M*_*4*_	*CuA*	*?*	*CuP*	*A*_*1*_	*A*_*2*_
*Schubnel (2021)*	*C*	*ScP*	*RA*	*RP*	*RP*	*MA*	*MP*_*1*_	*MP*_*2*_	*MP*_*3*_	*MP*_*4*_	*CuA*	*CuP*	*fiv*	*PCu* + *A*_*1*_	*A*_*2*_
Schmitz (1929)	C	Sc	R_1_	R_S_	R_2+3_	R_4+5_	M_1_	M_2_	/	CuA_1_	CuA_2_	/	A_1_ + CuA_2_	/	/
McAlpine and Martin (1966)	C	Sc	R_1_	R_S_	R_2+3_	R_4+5_	M_1_	M_2_	/	CuA_1_	/	/	A_1_ + CuA_2_	/	A_2_
Grimaldi (1989)	C	Sc	R_1_	R_S_	R_2+3_	R_4+5_	M_1_	M_2_	/	CuA_1_	/	/	/	A_1_	A_2_
Disney (1994)	C	S	1	R	2	3	4	5	/	6	/	/	7	/	/
Disney (2001)	C	S	R_1_	R_S_	R_2+3_	R_4+5_	M_1+2_	M_3+4_	/	A_1_	/	/	A_2_	/	/
Brown et al. (2015)	C	Sc	R_1_	R_S_	R_2+3_	R_4+5_	M_1_	M_2_	/	M_4_	CuA	/	CuA + CuP	/	A_1_
**New model**	**C**	**Sc**	**RA**	**RP**	**RP**_**1+2**_	**RP**_**3+4**_	**M**_**1**_	**M**_**2**_	**M**_**3**_	**M**_**4**_	**CuA**	**CuP**	**fiv**	**PCu** ** + A**_**1**_	**A**_**2**_

The homogenisation of the wing vein terminology presented here is the result of the observations of many fossil specimens and the analytical literature research on the wings of Diptera with emphasis on Phoridae. In this study, we present a new interpretation of the wing terminology through the creation of two models—an early and a recent one—that represent the majority of the wing vein diversity found on the fossil and living species. Within the *recent model*, we also added an example of a wing with extreme vein reduction. We applied this new terminological interpretation to three Phoridae holotypes, which were examined and re-evaluated: †*Euliphora grimaldii* Arillo and Mostovski, †*Prioriphora schroederhohenwarthi* Solórzano-Kraemer and Perrichot, and †*Ulrichophora lobata* Brown. In addition, we described two isolated wings, one Phoridae and one Ironomyiidae, representing the first records of these families in the respective Cretaceous deposits, Fouras-Bois Vert amber (included in Charentese amber, France), and San Just amber (Spain).

## Results

The study of the wings of the fossil has evidenced: (1) a low number of species described in contrast to the fossils discovered and, for those described, limited morphological information is provided; (2) sexual dimorphism occurs in the modern fauna but not in the fossil record; (3) high variability of interpretations in the wing vein terminology; and (4) the need to create standardised wing models and a new proposal of primary homology of venation in phorids.

### Number of extinct species described

**Fig. 1:**
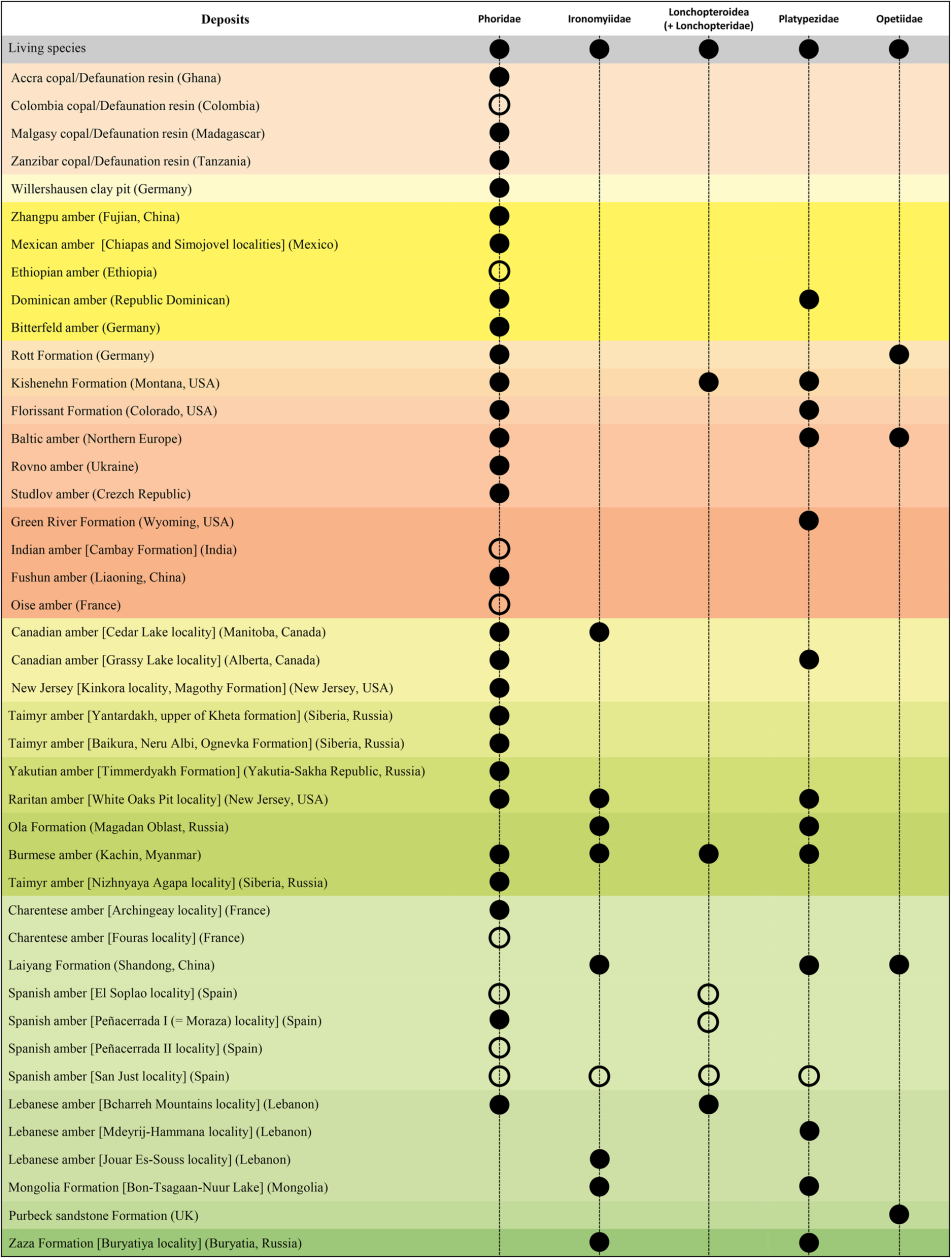
Overview of Phoridae, Ironomyiidae, Lonchopteridae, Platypezidae, and Opetiidae in fossil resins. Filled black circles, at least one species has been described in the literature. Empty black circle, no species has yet been described, but there are known specimens in collections

Only 307 specimens of fossil Phoridae have been studied so far. These belong to 119 described species in 41 genera (23 are extinct genera), 68 of them in Cretaceous amber, belonging to 22 species and 15 genera. Moreover, 82.1% of these were published before 2000, and only 21 species have been described in the last 24 years. Closely related families are much less abundant in the fossil record, such as the Platypezidae or the Ironomyiidae, which have 26 described species belonging to 17 genera (15 are extinct genera) and 28 described species belonging to only six genera (all are extinct genera), respectively. The Lonchopteroidea (including Lonchopteridae) have five described species belonging to four genera (three are extinct genera) and the Opetiidae have six species belonging to four genera (three are extinct genera) (Fig. 1).

### Sexual dimorphism

To date, no Cretaceous female phorid with a significant reduction of wing venation has been reported. In addition, the Cretaceous specimens examined for the present work, from various deposits, show no significant dimorphism and both have well-developed wing venation. However, it is important to note that only a few species in the amber fossil record are preserved together in a single piece representing both sexes.

### Variability of interpretations in the wing vein terminology and new models

**Fig. 2:**
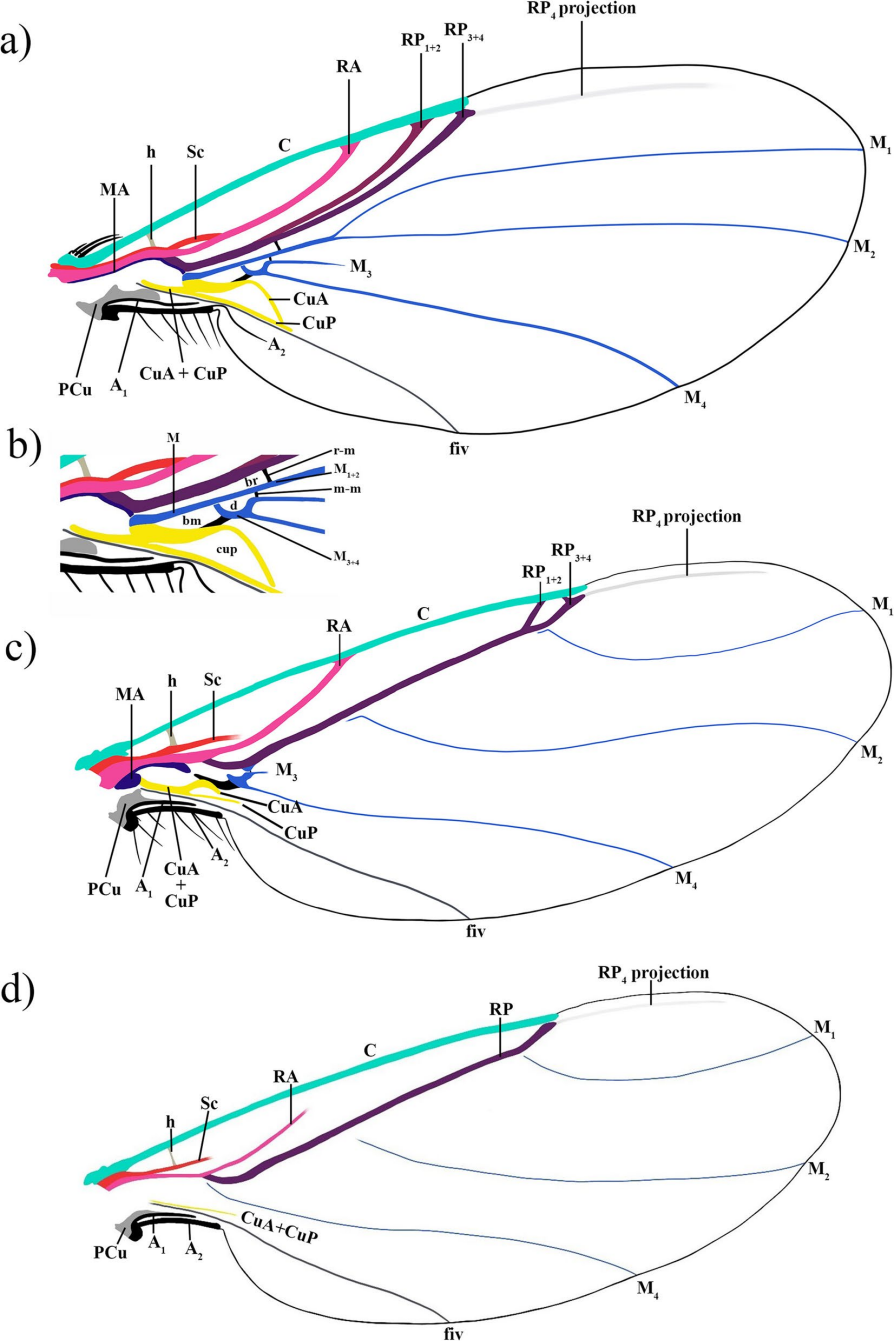
Representation of the new ground-plan wing venation models of Phoridae with new combined terminology. **a** and **b** represent the wing venation of the *early model* based on fossil specimens: **a** full wing; **b** proximal part of the wing. And **c** and **d** represent the recent wing venation: **c** the *recent model* based on modern specimens; **d** an example of a *recent model* with an important reduction of wing venation

Homogenisation of wing vein terminology of the phorids is critical for understanding the evolutionary history of this family over time. In an effort to standardise the various terminologies used in the study of the family Phoridae *sensu lato*, which includes “sciadocerines”, and in the study of the related families, two models have been established: an *early model* and a *recent model* (Fig. 2a,c), which serve as a valuable tool for describing both extinct and extant specimens. These models aid in distinguishing between species with more wing veins and those with a significant reduction in venation. The *early model* is derived from the wing venation pattern observed in Cretaceous specimens, while the *recent model* is based on species that emerged after the Cretaceous period, with a reduced wing venation pattern. An example of the *recent model* with even more reduced veins has also been made, to assist with the labelling of phorid wings with significant vein reduction (Fig. 2d). To achieve this, we have conducted a comprehensive investigation that combines the analysis of a large number of phorid wings from the existing literature with a thorough re-examination of selected extant specimens and the study of Cretaceous fossil specimens. The result of all these observations has led to a new interpretation of wing veins that applies to the entire family (Table 1).

**Fig. 3:**
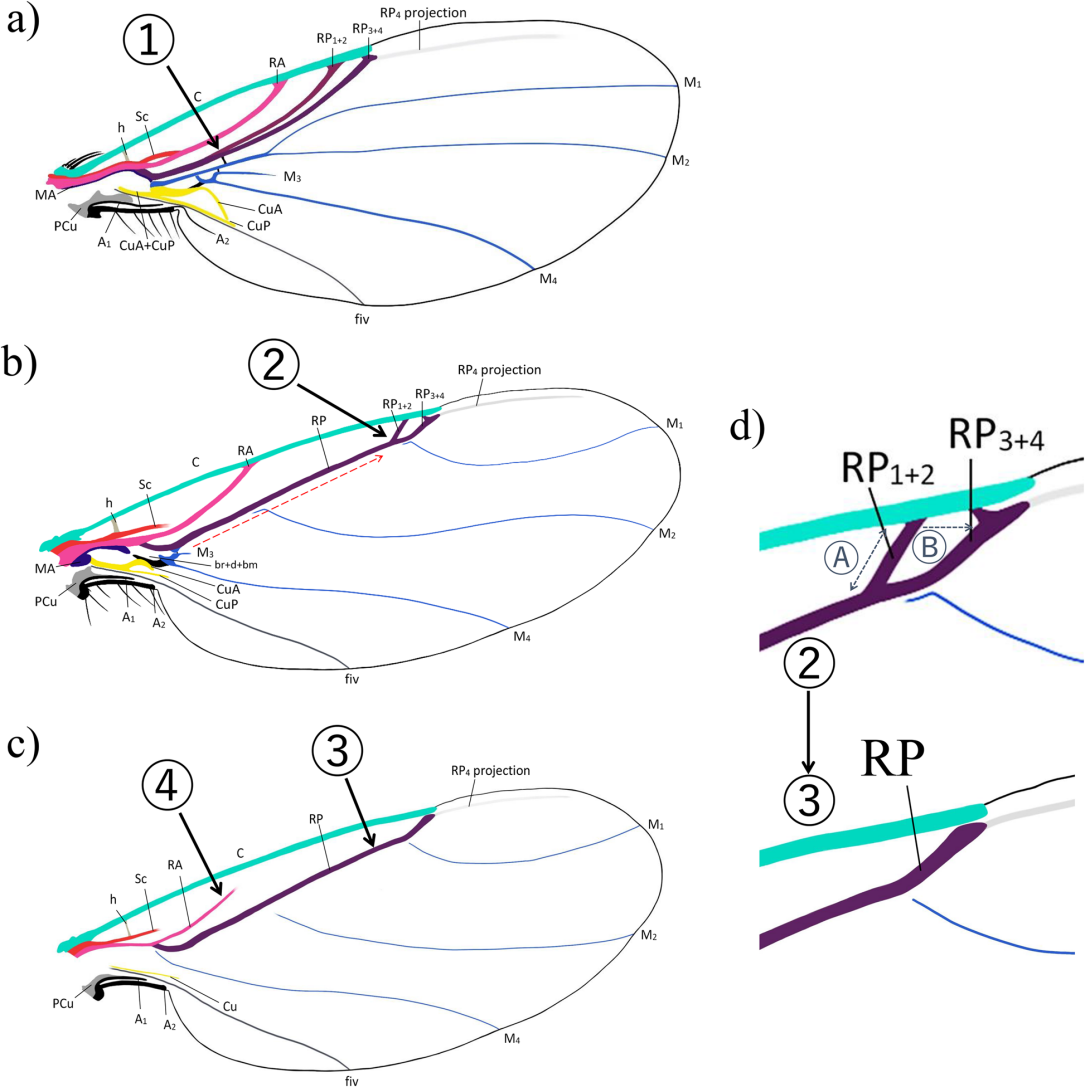
Hypothesis of reduction of Phoridae radial veins. **a** Proximal wing, long fork of RP fork ①. **b** Recent wing, short fork of RP vein ②. **c** Wing with an important reduction of venation, lost fork of RP vein ③, and distal reduction of RA vein ④. **d** Zoom of possibility of reduction of RP_1+2_, lost of RP_1+2_ vein either distally or proximally Ⓐ, or by the fusion of RP_1+2_ and RP_3+4_ veins Ⓑ

The establishment of this new terminology interpretation of Phoridae wing veins (Fig. 2 and Table 1) is further supported by comparison with other terminologies of dipteran wing venations such as Comstock and Needham [17], McAlpine et al. [8], Wooton and Ennos [18], Chapman [19, 20], Saigusa [21], Cumming and Wood [13], and Schubnel [16], and the different terminologies of phorid wing venation from Schmitz [26], McAlpine and Martin [28], Grimaldi [23], Disney [1, 31], and Brown et al. [3] (Table 1). The revision of the wing veins recognises that the radial veins have a distal division, with vein R bifurcating into two branches, namely RA and RP as in the interpretation of Schubnel [16], modified after the general wing venation model of Kukalová-Peck [32]. The RA commonly called R_1_ for Diptera is the anterior branch of the radial vein. The RP is the posterior branch of the radial vein commonly referred to in the literature as Rs and is divided into RP_1+2_ (in literature known as R_2+3_ or Rs_1+2_) and RP_3+4_ (known as R_4+5_ or Rs_3+4_). Some modern genera, e.g. *Chonocephalus* Wandollek, *Postoptica* Disney, *Triphleba* Rondani, *Phora* Latreille, and *Conicera* Meigen show only two branches of the radial vein. The current data were inadequate for the resolution of the last modification or modifications of the RP vein reduction (Figs. 2 and 3). Thus, to prevent different interpretations of the reduction, disappearance, or fusion of the second radial vein, we have chosen to maintain one anterior and one posterior radial vein, a feature consistently found in all Phoridae species (Figs. 2 and 3; Table 1). This approach ensures homogenisation of terminology across the family.

Chapman [20] proposed the division of the medial veins into anterior and posterior, but when MA cannot be distinguished, he proposed to use only medial veins. Schubnel [16] also separates the medial veins into the anterior and posterior, but he does not make a distinction when anterior or posterior is absent. However, these suggestions are not followed here because the presence or absence of MA is not homogeneous across all Phoridae. In some Phoridae genera, the MA is present but very difficult to observe (e.g. some species of *Myriophora* Brown, *Phora*, *Chaetopleurophora* Schmitz, *Sciadocera* White, *Archiphora* Schmitz). Nevertheless, in the majority of Phoridae genera, this vein is absent (e.g. *Anevrina* Lioy, *Chonocephalus*, *Darwiniphora* Schmitz, *Triphleba*). When MA is present, it is a short vein running along R ending before R divides into two distinct branches (RA and RP). The remaining medial veins retain the name M and are divided into M_1+2_ and M_3+4_, which in turn further subdivide into M_1_, M_2_, M_3_, and M_4_ (Figs. 2 and 3). The classification of the M_4_ vein is a subject of debate in the literature, primarily due to reduced wing venation and its lack of connection to the proximal vein, with various interpretations depending on the authors. The M_4_ is also referred to as CuA_1_ [8] and used in phorid descriptions (e.g. [8, 23, 26]). The revision of the literature and new specimens of various Cretaceous deposits demonstrate that M_3_ is present as a long vein (e.g. †*Prioriphora* n. sp.) (Fig. 2a,b), a short vein (e.g. †*Euliphora* Arillo and Mostovski) (Fig. 2c), or as an absence of microtrichia in the zone between M_2_ and M_4_ (e.g. *Chonocephalus*, *Trophithauma* Schmitz) (Fig. 2d). M_3_ in the referred genera is connected with the third long M vein, which has had a lot of labelling (i.e. CuA_1_, MP_2_, vein 6, A_1_; see Table 1), herein, thus this third long M vein is considered as M_4_.

The observations of the posterior part of the wing fit the interpretation of Schubnel [16]. The CuA vein is short and posteriorly curved. The CuP is predominantly transparent and can be difficult to observe and is often confused with the intercalary vein (i.e. the fiv). The fiv is thin and runs either beneath or along the CuP vein. Proximally, fiv connects to the proximal cell and extends distally to the wing edge. The fiv is not fused with other veins and is referred to in the literature by a variety of terms: A_1_, A_1_ + CuA_2_, A_2_, CuP, CuA + CuP (Table 1). The PCu vein is observed in the wing of Cretaceous specimens and early modern phorids, but it is absent in the majority of modern phorid species with reduced wing venation. The A_1_ vein is difficult to distinguish, runs along the posterior side of the PCu vein, and ends in the alular part of the wing. The A_2_ runs along the edge of the alula and sometimes extends into the anal part. For the terminology of cells and crossveins, we decided to keep and follow Cumming and Wood’s [13] nomenclature.

### Description of the veins in the two wing models

#### Costal vein

In both models, the tubular C is variable in length and usually ends before the wing apex at the level of RP_3+4_ (Additional file 1: Figs. S1c; S2b). However, in rare species, such as *Anevrina* sp. or *Postoptica continentalis* Lengyel and Papp, C continues slightly beyond RP_3+4_. In some others, such as *Neopleurophora kungi* Ament and Amorim, *Termitophilomyia* Schmitz, or *Javanoxenia* Schmitz, C is shorter than RP ([33]: figs. S25, S27, S33).

#### Subcostal vein

**Fig. 4:**
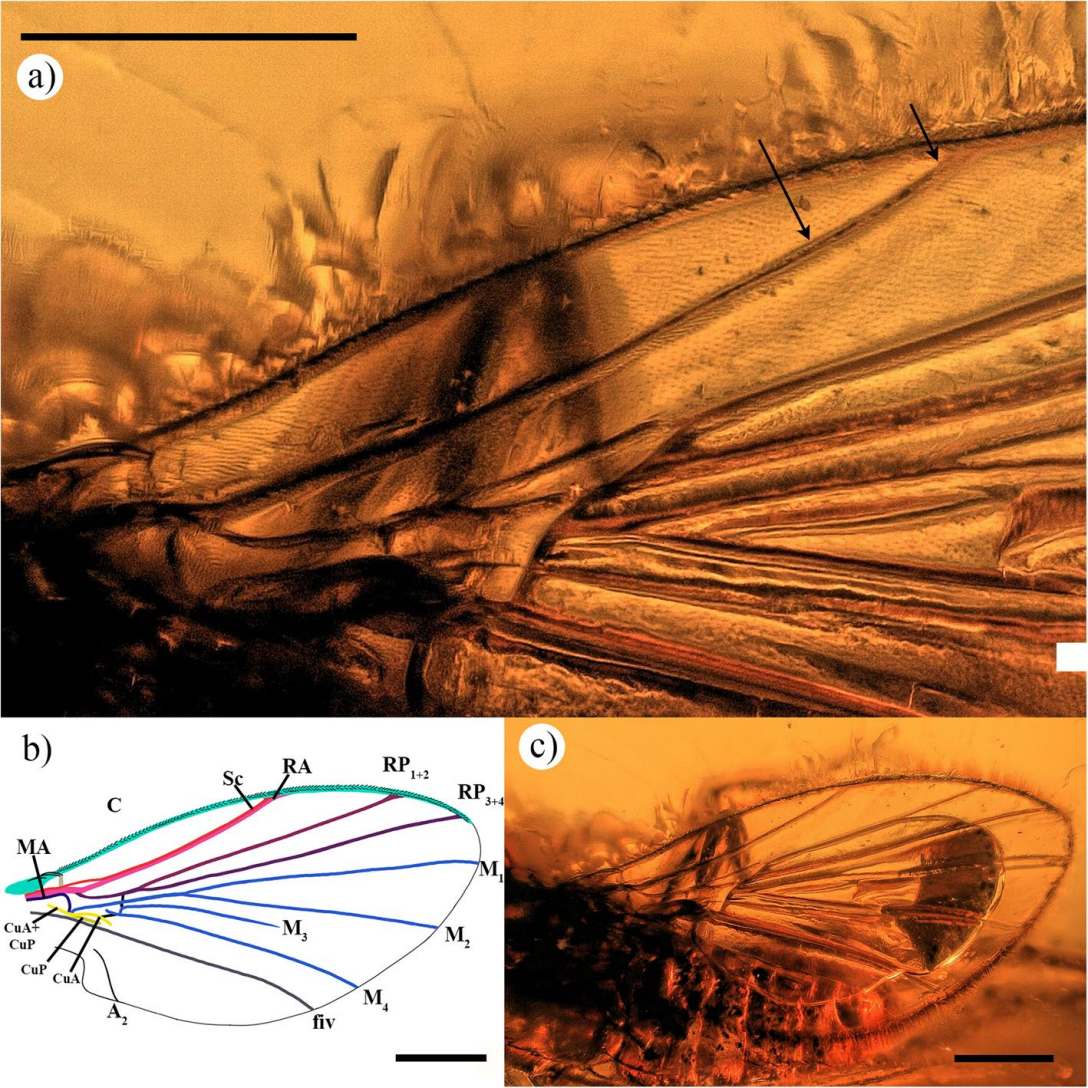
Wing of †*Ulrichophora lobata*, holotype LACM ENT 159890. **a** Zoom of the base of the left wing. **b** Drawing of the left wing. **c** Complete left wing. Scale 0.5 mm

In the *early model*, the tubular Sc is short and fuses with the RA at mid-length in most cases (Fig. 2a) such as †*Agaphora iunior* Mostovski (Additional file 1: Fig. S3), except for †*Ulrichophora lobata* (Fig. 4a). In †*U. lobata*, the Sc does not fuse directly with the RA; instead, it runs towards the RA and eventually fuses with the C. However, it is important to note that in †*U. lobata* the Sc does not diverge from the RA distally, unlike the Ironomyiidae. In the *recent model*, the Sc is shorter and gradually ends vanishing before reaching the RA (Additional file 1: Figure S2; [33]: figs. S25–S26).

#### Radial veins

**Fig. 5:**
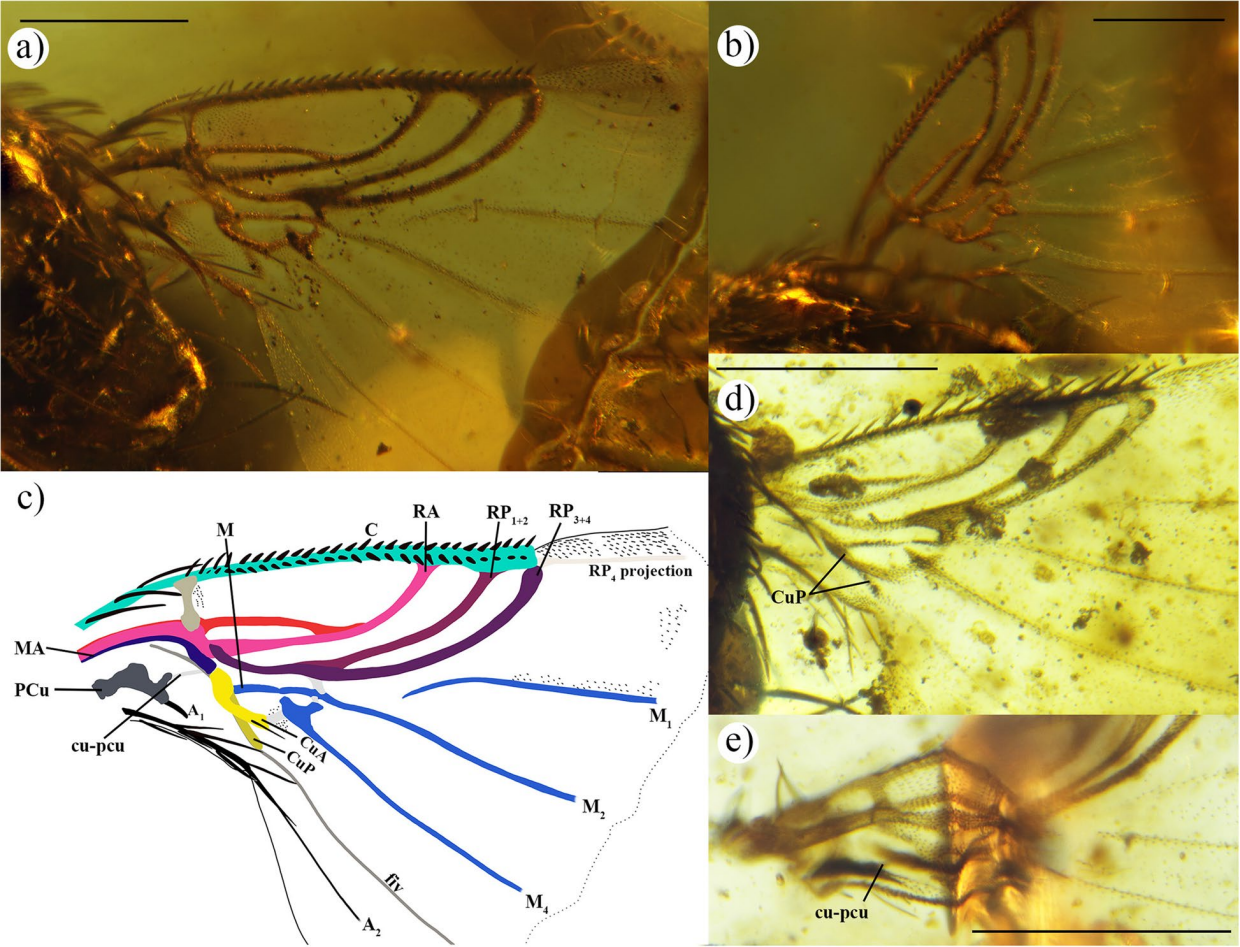
Wings of †*Euliphora* species.** a–c** Wing of †*Euliphora grimaldii*, holotype MCNA 8648: **a** proximal part of the left wing; **b** proximal part of the right wing; **c** draw of the left wing; **d** right wing of †*Euliphora* n. sp. 1, MCNA 12734, Álava (Peñacerrada I); **e** isolate left wing of †*Euliphora* n. sp. 2, MCNA 15469, from Álava (Peñacerrada I) distorted in proximal. Scales 0.2 mm

The tubular R is divided into the RA and RP after the h crossvein. Typically, the tubular RA fuses with the C, except in a few species such as *Postoptica continentalis*, or *Chonocephalus fletcheri* Schmitz, in which the RA vanishes before reaching C (Additional file 1: Fig. S2). In the *early model*, the tubular RP undergoes a proximal division into the RP_1+2_ and RP_3+4_, which occurs either slightly before or after the r-m crossvein such as †*Agaphora iunior* (Additional file 1: Fig. S3). This division results in the formation of a long fork (Figs. 2a and 3a). The tubular RP_1+2_ fuses with the C, while the tubular RP_3+4_, does not. However, in a few species such as †*Ulrichophora lobata* (Fig. 4), *Sciadocera rufomaculata* White; and *Archiphora patagonica* Schmitz, the RP_3+4_ fuses with C (Additional file 1: Fig. S1); and thus, it is considered as a distinguishing character of the Sciadocerinae. In the *recent model*, the RP exhibits a distal division into two branches, forming a short fork (Figs. 2c and 3b,d; Additional file 1: Fig. S2a). This character is observed in taxa such as *Dohrniphora* Dahl, *Kuenburgia* Schmitz, *Megaselia* Rondani, and *Spiniphora* Malloch. In certain taxa (e.g. *Aenigmatistes* Shelford, *Conicera*, *Gymnoptera* Lioy, *Phora*), the RP is undivided (Figs. 2d and 3c; Additional file 1: Fig. S4b,c); in such cases, it does not fuse with the C. The RP_1+2_ may fuse with the C, or if it is unfused, but the veins are much more distally forked; this can be observed in a few species such as *Chaetopleurophora rhomboidea* Nakayama. In some instances, a portion of the RP_1+2_ remains fused to the C, but is no longer attached to the RP, as observed in species like *Coniceromyia riccardiae* Ament, Kung and Brown or *Coniceromyia strongyla* Ament, Kung and Brown. The RP_3+4_ does not fuse with the C. In both models, the presence of the longitudinal RP_4_ varies. In cases where the RP_4_ projection is present, it may manifest as a thin vein or as a vestigial or spectral projection vein. This can be distinguished by a parallel line with the anterior wing margin, often represented by a line of concentrated microtrichia or a darker-coloured line on the wing surface (Figs. 2 and 5; Additional file 1: Figs. S2; S4a; S5).

#### Medial system (veins and crossveins)

The medial veins differ greatly between the *early* and *recent models*. The MA may be present in both models. If present, it starts from the base of the wing, is relatively narrow, and extends along the R to the base of the RP (Figs. 2a and 3a). However, it may be completely absent. In the *early model*, the r-m crossvein is located either at the same level or slightly beyond the RP fork and at the same level or slightly beyond the m-m crossvein (Fig. 2b). In the *recent model*, r-m crossvein is mostly absent (Fig. 2d).

In the *early model*, the M is present and splits into the M_1+2_ and M_3+4_ (Fig. 2a,b), but M_3+4_ is not connected to M (Fig. 2b). The M_1+2_ splits into M_1_ and M_2_ beyond the RP fork (Fig. 2a,b). The M_1_ is connected to M_1+2_ in some cases but in others, M_1_ is spectral at its proximal part (Additional file 1: Fig. S6a). The M_2_ is connected or not to M_1+2_. The M_3+4_ is short and slightly swollen and splits into the M_3_ and M_4_ behind the m-m (known as dm-m) crossvein (Fig. 2b; Additional file 1: Fig. S1b). The M_3_ is rather infrequent, short, narrow, and rapidly disappearing (Fig. 2a), or present only as a short vestigial vein. The M_4_ usually reaches the wing margin, but sometimes disappears just before. The M_1_, M_2_, and M_4_ are always posteriorly curved (Additional file 1: Fig. S1a).

In the *recent model*, the M is absent (Fig. 2c). The M_1+2_ is represented by a narrow vein running closely along the RP or as a short remnant vein at its base (Fig. 2c), or the vein is completely absent (Fig. 2d). In any case, it is difficult to observe. The bifurcation of M_1+2_ into M_1_ and M_2_ is not visible. The M_1_ and M_2_ are straight, anteriorly curved, or proximally anteriorly curved and distally posteriorly curved such as in †*Agaphora iunior* (Additional file 1: Figs. S3; S6b). The M_1_ never fused with RP. The M_1_ may run a little along the RP reaching RP_3+4_, or ends when the vein is very close to RP_3+4_ (Fig. 2c; Additional file 1: Fig. S6b). However, for a few species such as *Chonocephalus* sp. or *Metopina* sp. (Additional file 1: Fig. S2c,d), the proximal part of M_1_ is reduced and does not reach RP_3+4_ but conserves the direction towards RP_3+4_ (Fig. 2c). The proximal part of M_2_ is vanishing and runs slightly along RP or ends near RP about halfway between the R fork and RP fork or the end of RP if the RP fork is absent (Fig. 2c). The M_3+4_ is represented by a swollen short vein (Fig. 2c), or it is completely absent (Fig. 2d). The M_3_ can be absent or present, sometimes also represented by a line without microtrichia (e.g. *Coniceromyia megalosoma* Ament, Kung and Brown, or *Metopina* sp.) (Additional file 1: Fig. S5c). The M_4_ is straight, posteriorly curved, or slightly anteriorly curved, derived from the M_3+4_; however, if the M_3+4_ is absent, the M_4_ is vanishing proximally such as in *Chonocephalus* sp. (Additional file 1: Fig. S2d).

The longitudinal m-m crossvein is located between the M_1+2_ and M_3_ (Fig. 2b). It is a small tubular crossvein, almost transparent, and sometimes difficult to observe when the M_1+2_ and M_3_ are close together. The crossvein m-cu is present; it is more-or-less long and is located between the M_3+4_ and the CuA (Fig. 2b); but if both veins are absent, m-cu is also completely absent (Fig. 2d). Crossveins m-m and m-cu are present in the *early model*, whereas the crossveins m-m are absent and m-cu are present or absent in the *recent model.*

#### Proximal cells

In the *early model*, br, bm, and d (known as dm, e.g. [1, 3]) cells are represented through the proximal medial system (Fig. 2b; Additional file 1: Fig. S6a). The length of the br cell varies depending on the position of r-m, with it either opening proximal or being enclosed by the MA. The bm and d cells are not merged but are separated by the M_3+4_, with the d cell slightly opening towards the distal side to bm. The boundary between br and bm is defined by the M, while the separation between br and d is marked by the M_1+2_. In the *recent model*, there is a reduction of the proximal part of wing veins (Fig. 2c,d). There are three observable cases. First, the two cells br and bm + d are present. M and M_1+2_ are present, br cell present but M_3+4_ absent, bm and d cells are fully open and form a unique cell bm + d. Second, only one cell br + bm + d is present. This is the result of the proximal reduction of medial veins. M, M_1+2_ and M_3+4_ are absent, but the surrounding veins are present, bounded anteriorly by a base of radial s, distally by the medial veins and the m-m crossvein, and posteriorly by the cubital veins and the m-cu crossvein. Third, all cells are absent. This is observable when the wing has an important wing vein reduction.

#### Cubital veins

In the *early model*, the CuA is short, distally posteriorly curved, and dips rapidly towards CuP (Fig. 2a). If CuP is reduced or absent, CuA dips towards the fiv at the level of m-cu crossvein. The CuA is mainly spectral before joining fiv. In the *recent model*, the cubital veins are present or absent (Fig. 2c,d); if they are present, the CuA and CuP are shorter than in the *early model*. The CuA is distally posteriorly curved and reaches the CuP or the fiv just after m-cu crossvein, or it ends on the contact with crossvein m-cu. The CuP is represented by a spectral vein, usually transparent and difficult to observe. The CuP is distally vanishing before, or slightly after, the ends of the CuA, running closely along the anterior margin of fiv.

#### Intercalary vein

In the *early model*, the intercalary vein known as fiv is present. It appears as a narrow vein located between the alula and the CuP in the anal region of the wing. The fiv does not connect another vein, from the proximal cell of the wing, but runs proximally closely along the posterior margin of the CuP until it reaches the wing margin (Fig. 5a,c; Additional file 1: Fig. S1). In the *recent model*, when proximal cells are present, the fiv maintains a similar configuration to that of the *early model*. However, when the proximal cells are absent, the proximal fiv either vanishes and terminates just before the wing margin or is completely absent.

#### Postcubital

In both models, the PCu may be present or absent. The PCu is short and wide (Additional file 1: Fig. S1b), with one or two branches [22].

#### Anal veins

The A_1_ is short, ends in the alular part of the wing, and is difficult to distinguish from the PCu. The A_1_ runs closely along the posterior margin of the PCu (Fig. 2). The PCu and the A_1_ appear to be fused. In the *early model*, the A_2_ is long, runs along the margin of the alula and continues into the anal part of the wing (Fig. 2a). In the *recent model*, the A_2_ is short and ends in the alula, or it is absent (Fig. 2c,d).

#### Comments

The majority of Cretaceous species show characters fitting only the *early model*, with the exception of †*Agaphora* Mostovski species and †*Metopina goeleti* Grimaldi. The †*Agaphora* species have a wing pattern that fits with both models, an early radial system and a recent medial system. We refer to this group as the “Agaphorine-group”. The species †*M. goeleti* has a reduced wing pattern, with a reduced radial and medial system as in the *recent model*. The †*Agaphora* species and †*M. goeleti* have not been revised; thus, the characters discussed here are exclusively from the literature. Only two Tertiary species from the Baltic amber (†*Ulrichophora lobata* and †*Eosciadocera pauciseta* Grimaldi) and two living species (*Archiphora patagonica* and *Sciadocera rufomaculata*) also show characters fitting the *early model*. These four species are assigned to the subfamily Sciadocerinae. However, the re-evaluation of †*U. labata* shows a Sc vein that does not fused in RA, unlike all the other species. This feature is also found in the wing pattern of the family Ironomyiidae, but with the difference that distally, in †*U. lobata*, the Sc does not separate from the RA before merging with C. All other Cenozoic and living species have reduced veins that fit with the *recent model*.

#### Outgroup comparison

For the four families Lonchopteridae (Additional file 1: Fig. S7a), Opetiidae (Additional file 1: Fig. S7b), Platypezidae (Additional file 1: Fig. S7c), and Ironomyiidae (Additional file 1: Fig. S7d): the costal vein stops at the tip of wing; Sc vein and all the radial veins fuse to C. The Lonchopteridae and Opetiidae are closed, the medial veins system is similar with a long fork of M_1_ and M_2_ and absence of d cell and m-m crossvein. The wing patterns of Platypezidae and Ironomyiidae are closer than those of Phoridae, the medial vein system is similar with a large d-cell and the presence of a long m-m crossvein.

### Systematic palaeontology

Order Diptera Linnaeus, 1758.

- Family Phoridae Curtis, 1833
- Genus †*Euliphora* Arillo and Mostovski, 1999
- Type species: †*Euliphora grimaldii* Arillo and Mostoski, 1999: 252–254, figs. 1–4.
- Monogeneric.

†*Euliphora grimaldii *Arillo and Mostoski, 1999 (Fig. 5a–e)

Material examined: Holotype, ♂, SPAIN: Álava, Albian, Lower Cretaceous, Álava amber (or Peñacerrada I, Spanish amber), amber piece MCNA 8648 housed at MCNA (Museo de Ciencias Naturales de Álava).

Other specimens examined, not described in this study: Holotype, unknown, SPAIN: Álava, Albian, Lower Cretaceous, Álava amber (Peñacerrada I, Spanish amber) (MCNA 12734). Holotype, isolate wing, SPAIN: Álava, Albian, Lower Cretaceous, Álava amber (Peñacerrada I, Spanish amber) (MCNA 15469).

#### Revision and additional characters of holotype

Matching the *early model*. C covered with two rows of strong, short, spine-like setae. Sc long, runs slightly along the anterior margin of RA before fusing to RA. RA and RP_1+2_ fused to C, while RP_3+4_ not; RP_4_ continues spectrally parallel to the wing margin. MA runs along the posterior margin of R and ends at the level of R fork, and seems to run into CuA + CuP. Crossvein r-m present, broad at the level of RP fork. M present, M fork absent; M_1_ proximal vanishing before reaching M_1+2_; M_2_ proximal connected to M_1+2_; M_1_ and M_2_ mostly straight, and reach margin of wing; crossvein m-m narrow, tiny, and tubular; M_3+4_ short and strongly broad, proximal not connected to M; M_3_ present but as a short peduncle; M_4_ tubular; m-cu long. CuA + CuP short, broad, bulla-shaped; CuA ends just after m-cu connexion, with a seta posterior distally; CuP runs under CuA, and ends slightly after the end of CuA; cup cell absent. Intercalary vein, fiv, runs along the posterior margin of CuP, below CuA + CuP and slightly along MA before reaching the proximal cell of the wing; fiv proximally spectral and distally tubular. Crossvein cu-pcu present, long. PCu with two short branches. A_1_ proximally fused with the posterior branch of PCu. A_2_ long, runs along the axillary margin and extends into the anal part of wing. Eight setae on axillary ridge (instead of five setae mentioned in the original description).

#### Remarks

The characters “CuP runs under the CuA, and ends slightly beyond the CuA”, and “crossvein cu-pcu present, long” are not observed in other Cretaceous genera but observed in other specimens of Álava amber identified as belonging to the †*Euliphora* genus (†*Euliphora* n. sp. 1, Fig. 5d; and †*Euliphora* n. sp. 2, Fig. 5e), these specimens will be described in a future study. The crossvein cu-pcu connects the proximal part of the CuA + CuP vein to the proximal part of the PCu vein, and run under the fiv. These two morphological characters are considered diagnostic of the genus †*Euliphora*. No female of the genus †*Euliphora* has yet been found.

- Genus †*Prioriphora* McAlpine and Martin, 1966.
- Type species: †*Prioriphora canadambra* McAlpine and Martin, 1966: 532, figs. 2, 9–12, 21.
- Other included species: †*Prioriphora canadambra,* †*P. casei* Grimaldi and Cumming*,* †*P. cheburashka* Mostovski*,* †*P. longicostalis* Brown and Pike*,* †*P. luzzii* Grimaldi and Cumming*,* †*P. polyankae* Mostovski*,* †*P. schroederhohenwarthi,* †*P. setifemoralis* Brown and Pike.

**Fig. 6:**
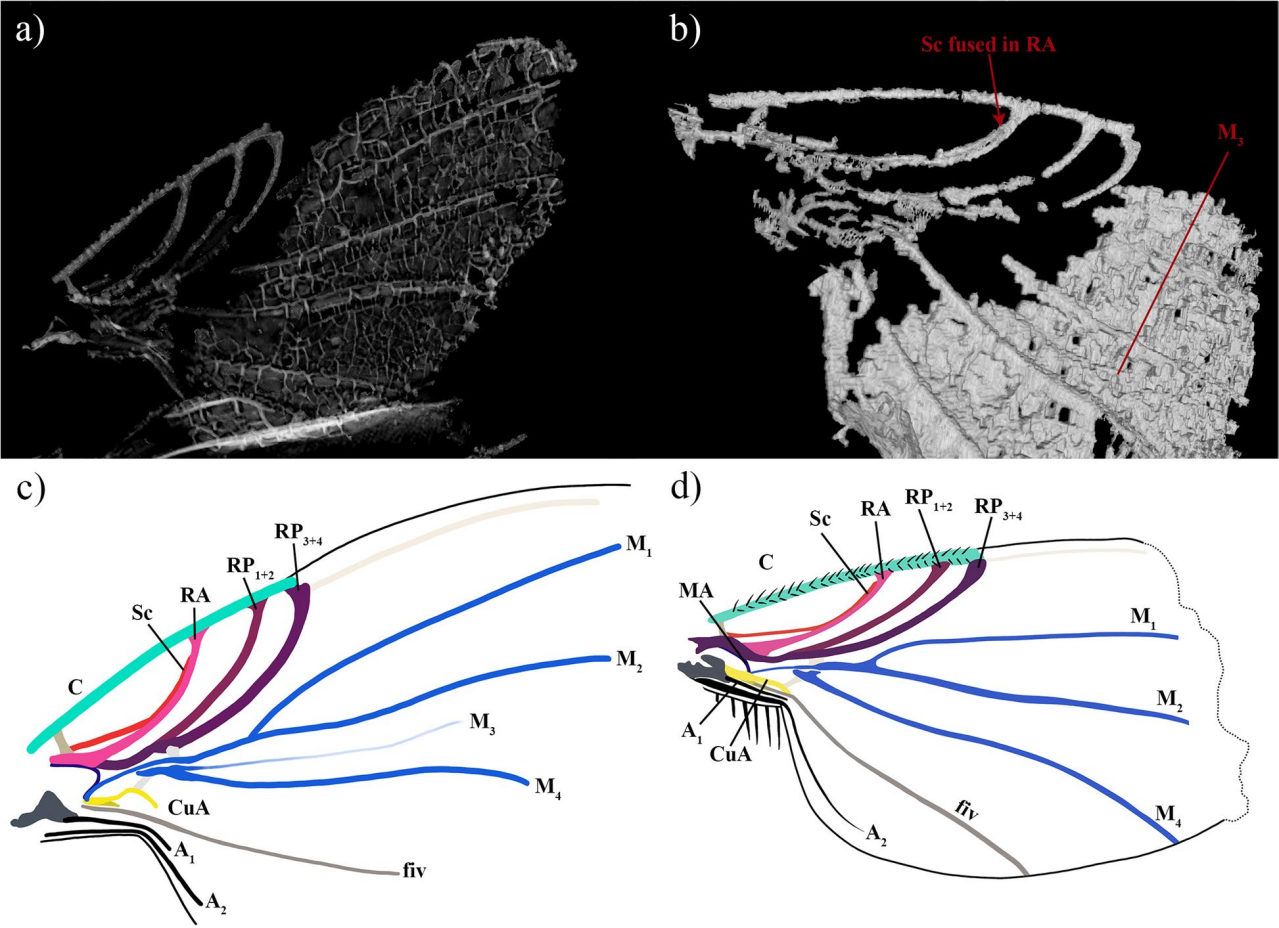
Wings of †*Prioriphora schroederhohenwarthi *Solórzano-Kraemer and Perrichot, holotype IGR-ARC-382. **a** Three-dimensional virtual extraction of the right wing ventrally. **b** Three-dimensional virtual extraction of the right wing dorsally. **c** Right wing drawing of the holotype. **d** Wing drawing of the paratype IGR-ARC-04

†*Prioriphora schroederhohenwarthi* Solórzano-Kraemer and Perrichot, 2011 (Fig. 6a–d)

Material examined: Holotype, ♂, FRANCE: Charente-Maritime, Archingeay, Cenomanian, mid-Cretaceous, Archingeay amber (Charentese amber), amber piece IGR-ARC-382.1b housed at IGR (Geological Institute of Rennes). Paratype, isolate wing, FRANCE: Charente-Maritime, Archingeay, Cenomanian, mid-Cretaceous, Archingeay amber (Charentese amber) (IGR-ARC-04).

#### Revision and additional characters of holotype

Matching the *early model*. C with two rows of strong short, spine-like setae (instead of “covered with minute setae” mentioned in the original description); C_ratio_: 4.3; 1.5; 1. Sc runs along RA and fused to RA just before RA fused to C (Fig. 6). RA and RP_1+2_ fused with C, while RP_3+4_ not; RP fork long; RP_4_ continues spectrally parallel to the anterior margin of wing. Cossvein r-m broad, at level of RP fork. MA long, runs along R and before R fork level plunges towards in proximal CuA + CuP, distally seems to fused with proximal tip of CuA + CuP and M. M and M_1+2_ present; M seems to originates from MA and CuA + CuP; M_1+2_ divides into M_1_ and M_2_; M_1_ and M_2_ slightly straight, and reach margin of wing; crossvein m-m tiny and tubular; M_3+4_ vanishing, but short and wide, not connected to M; M_3+4_ divided in M_3_ and M_4_; M_3_ present, short proximal growth, then by an absence-of-setae line in the paratype (e.g. observation in paratype photograph in [34]: fig. 4c), and observable by a long remnant vein in the holotype (Fig. 6b). Cells br, bm and d, present; br cell elongates and narrow, proximal part close by MA and M; proximal part of bm cell close by M and CuA + CuP; d cell tiny, and slightly open to bm cell, distally close by crossvein m-m. CuA + CuP present; CuA ends quickly after crossvein m-cu; CuP short, vanishing before connection of CuA with m-cu; cup cell absent (instead of cup cell present and “anterior margin of cell cup fused to Rs, A_1_ + CuA_2_ runs into posterior margin of cup” mentioned in the original description). Proximal section of fiv seems to vanish at proximal tip of CuA + CuP, then runs along posterior margin of CuP, and reaches posterior wing margin. PCu with two short branches. Proximal part of A_1_ fused with posterior branch of PCu; A_1_ long, and ends quickly in the anal part of wing. A_2_ long, runs along axillary margin and extends into the anal part of wing. Seven setae on axillary margin.

**Fig. 7:**
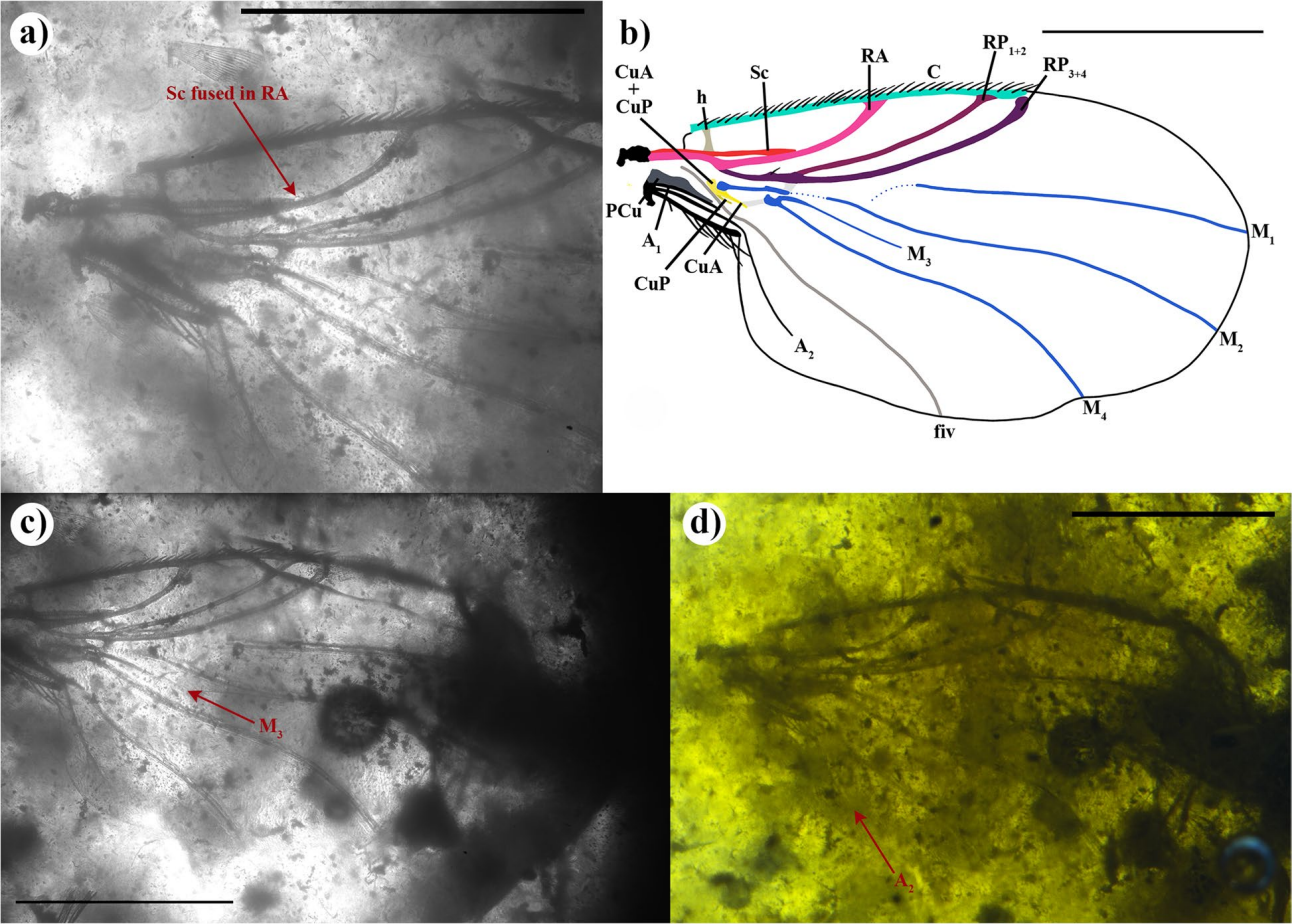
Wing of †*Prioriphora* n. sp., collection number IGR-FRS-7.** a** and **c** Infrared reflected photographs of the left wing: **a** zoom of the base of the wing, **c** complete wing, **b** drawing of the left wing, **d** a photograph of the left wing. Scale 0.5 mm

†*Prioriphora* n. sp. (Fig. 7a–d)

Material examined: Holotype, isolate wing, FRANCE: Charente-Maritime, Fouras, Cenomanian, mid-Cretaceous Fouras-Bois-Vert amber (Charentese amber) (IGR-FRS-7). The amber piece IGR-FRS-7 contain several inclusions, 53 arthropods and 3 conifers fragments, the list of syninclusions detailed in [35].

#### Diagnosis

Sc runs slightly along RA before fused with RA; distal RP with one seta; RP_3+4_ slightly longer than C. RP_4_ projection and MA absent. M_3_ present, long, and proximally tubular before vanishing in three-quarters of M_4_ length. A_2_ long and separated to axillary ridge.

#### Description of wing

Matching the *early model*. Wing length 1.14 mm and width 0.70 mm. Wing covered with minute setae. C long; CI: 0.64; C_ratio_: 4.1; 2.8; 1; C covered by two rows of dense, medium, spine-like setae. Sc fused to RA in the mid-length. RA and RP_1+2_ fused with C, while RP_3+4_ not; RP fork long. Cossvein r-m broad, at level of RP fork. M and M_1+2_ present; M seems to connect with CuA + CuP; M_1+2_ extends to M_2_, fork absent; proximal M_1_ vanishes well before reaching M; M_1_ straight and distally posteriorly curved while M_2_ sigmoid; M_1_ and M_2_ reach margin of wing; crossvein m-m tiny and tubular; proximal M_3+4_ vanishing, not connected to M; M_3+4_ short, strong, and wide, divided in M_3_ and M_4_. Cells br, bm and d, present; br cell elongates and narrow, proximal cell open in the proximal cell; proximal bm cell close by M and CuA + CuP; d cell small, and slightly open to bm cell, distally closed by crossvein m-m. CuA + CuP present; CuA ends at level of crossvein m-cu; CuP short, transparent, vanishing quickly; cup cell absent. Proximal tip of fiv starts from the middle of the proximal cell, then runs along the posterior margin of CuA + CuP and CuP; fiv reaches the posterior wing margin. PCu with one branch. A_1_ short, runs along posterior PCu, and ends before the anal part of wing; A_2_ extends into the anal part of wing. Seven setae on axillary margin.

#### Remarks

As this is an isolated wing and no phorid body has been observed in the Fouras-Bois Vert amber, it is not possible to describe a new species. The wing veins are very similar to †*P. casei*, differing in that the fiv does not join the wing margin and no seem seta on RP are present (i.e. not mentioned in [36]: fig. 59); however, this wing differs from all other †*Prioriphora*. This wing is also very similar to the genus †*Gemmaphora* Mostovski, showing a similar CI and C_ratio_, but differing in the absence of the M_1+2_ fork (e.g. fork present is mentioned as a diagnostic character of †*Gemmaphora*). The genus †*Prioriphora* is rather problematic because of the five diagnostic characters of this genus, four are based on wing vein characters [37] that can also be found in other Cretaceous genera. Therefore, we have classified this specimen in the †*Prioriphora* genus. We decided to describe this wing for two reasons: (1) the particularity of the M_3_ which is long and whose proximal part is tubular, indicates that it is a new species of †*Prioriphora*; (2) the presence of Phoridae in a new locality reinforces the knowledge of the biodiversity of phorids in the Cretaceous and the possibility of finding new remains of phorids in the Fouras-Bois Vert amber in the future.

- Genus †*Ulrichophora* Brown, 2007.
- Type species: †*Ulrichophora lobata* Brown, 2007: 1–2, figs. 1–5.
- Monogeneric.

†*Ulrichophora lobata* Brown, 2007 (Fig. 4a–c)

Material examined: Holotype, ♂, RUSSIA: Kaliningrad, Baltic amber, amber piece LACM ENT 159890 housed at LACM (Natural History Museum of Los Angeles County).

#### Revision and additional characters of holotype

Matching the *early model*. Wing length 2.6 mm and width 1.22 mm. C long; CI: 0.95, and C_ratio_: 2.72; 1.63; 1 (i.e. paratype, [2]: figs. 4 and 5); C covered by two rows of tiny, dense, spine-like setae. Sc speedy runs along RA, but not fused to RA (black arrows in Fig. 4a), fused to C without separating from RA (instead of Sc fused to RA mentioned in the original description by “wing similar to that of extant sciadocerines”). RA, RP_1+2_, and RP_3+4_ fused with C not; RP long, and fork long. Crossvein r-m broad, between R fork and RP fork. M and M_1+2_ present; M seems to originate to proximal part of CuA + CuP; M_1+2_ fork present; M_1_ and M_2_ straight; M_1_ and M_2_ reach margin of wing; crossvein m-m longer than other Cretaceous species and tubular; proximal M_3+4_ vanishing, not connected to M; M_3+4_ short, as wide as other medial veins, divided in M_3_ and M_4_; M_3_ present, long, and proximally tubular before vanishing at mid M_4_ length. Cells br, bm and d, present; br cell elongates and large, proximal part close by MA and M; proximal part of bm cell close by M and CuA + CuP; d cell small, and slightly open to bm cell (in paratype, d cell close, [2]: figs. 4 and 5), distally close by crossvein m-m. Proximal section of fiv not close to CuA + CuP, then runs close along posterior margin of CuP, and reaches posterior wing margin. A_2_ long, runs along axillary margin and extends into the anal part of wing; holotype A_2_ reaches posterior wing margin while paratype A_2_ vanishes before reaching the wing margin. Seven setae on axillary margin.

Family Ironomyiidae McAlpine and Martin, 1966.

**Fig. 8:**
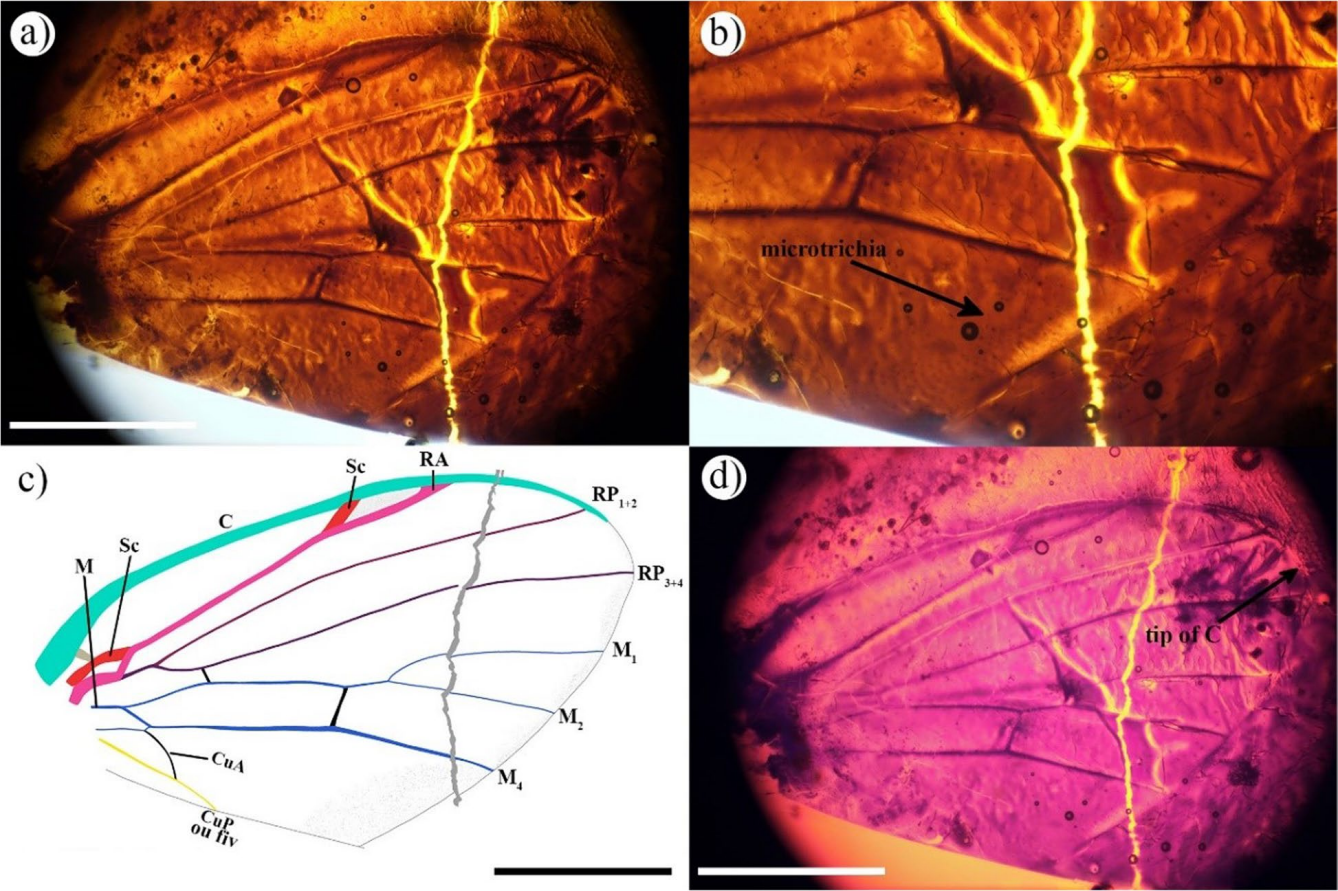
Wing of Ironomyiidae †*n. gen. *n. sp., collection number SJNB2012-12–10.** a** and **c** Wing photograph: **a** complete wing, **c** zoom of the base of the wing, **b** wing drawing, **d** infrared reflected photographs. Scale 0.5 mm

†n. gen. n. sp. (Fig. 8a–d)

Material examined: Holotype, isolate wing, SPAIN: Teruel, San Just, Utrillas municipality, Albian, Lower Cretaceous, San Just amber (Spanish amber) (SJNB2012-12–10). Proximal and postero-proximal wing part absent, the distal part is present but broken, in amber piece SJNB2012-12–10, with several inclusions, 36 arthropods, the list of syninclusions detailed in [38] and in “Additional file 2”.

#### Diagnosis

Wing hyaline except in the wing tip with a short microtrichia line between RP_3+4_ and M_4_ and a large microtrichia line after M_4_. C end between RP_1+2_ and RP_3+4_. Distal M_1+2_ part between m-m level and fork long; proximal M_1_ not in the continuity of distal M_1+2_; m-m slightly oblique.

#### Description of wing

Matching the *early model*. C setae minute and few dense. Sc fused to RA over most of their lengths. Cell d (length d/length wing) long 0.33.

#### Remarks

Wing is similar to †*Palaeopetia* Zhang, and †*Macalpinomyia* Li and Yeates in having a short microtrichia line between RP_3+4_ and M_4_ and a large microtrichia line after M_4_, and to *Ironomyia* White and †*Proironia* Grimaldi in being fully hyaline. In all other genera C end at or after RP_3+4_. †*Proironia* and †*Palaeopetia* also have minute setae along C, but in †*Palaeopetia* the setae are more dense. †*Palaeopetia* has Sc fused to RA over most of its length; however, in †*Macalpinomyia* Sc is not fused but is close to RA. †*Macalpinomyia* and †*Palaeopetia* are similar in having a long M_1+2_ between the crossvein m-m and the fork, however, in †*Proironia* this part is short and in *Ironomyia* and †*Cretonomyia* Li and Yeates absent. †*Macalpinomyia*, †*Palaeopetia*, †*Proironia* are similar in that the proximal M_1_ is not in continuity with the distal M_1+2_, but *Ironomyia* has it in continuity; †*Proironia* and †*Macalpinomyia* are similar in that m-m is slightly oblique; however, in *Ironomyia* m-m is strongly oblique, and perpendicular in †*Palaeopetia*. †*Macalpinomyia* and †*Eridomyia* Mostovsky are similar in having cell d long 0.33 (length d/length wing), in †*Palaeopetia* and †*Hermaeomyia* Mostovsky d is short [39]. Until now, no Ironomyiidae species were known from Spanish amber. The discovery of an Ironomyiidae in another Cretaceous deposit confirms their important biodiversity during this period while no Cenozoic fossils have yet been found during the Cenozoic (Fig. 1). The wing discovered differs from all currently described species in that it displays a new combination of characteristics not present in †*Macalpinomyia*, †*Palaeopetia*, and †*Proironia*. Together, all these features are an indication that this specimen would correspond to a new genus. Preservation of the wing is incomplete and in the absence of the rest of the body, we do not formally describe this specimen.

## Discussion

Describing fossil species in amber has been challenging across the centuries. Insects in amber are often in sub-optimal positions, the quality of preservation is sometimes poor, and together with the not-always-transparent nature of fossil resins limits our current knowledge of fossil insects, including phorids. Furthermore, the lack of morphological data from the fossil record is due to limitations such as inadequate microscopes or insufficient light intensity for detailed vein examination (i.e. the proximal part of the wing which is often obscured by the body, or impurities surrounding the specimen could hide the connections of veins). The scarcity of new descriptions is not due to the absence of fossil remains—thousands of phorid fossils have been discovered in various amber deposits, copal, and Defaunation resin, housed in worldwide museums and institutional collections—but several factors contribute to the relative unpopularity of the family. These include decentralised data, their small size, and the lack of extraordinary features that might captivate researchers, or else the unresolved family phylogeny which further discourages some new investigations. Additionally, many descriptions provide only minimal details [31, 40–48], sometimes with imprecise diagnostic characters, combined with the absence of an image or drawing making the use of these data for other studies complicated. In contrast, closely related families such as Lonchopteridae, Opetiidae, Platypezidae, and Ironomyiidae contain many more species described relative to the number of specimens found in various fossiliferous deposits. All these families have living representatives but interestingly, no specimens of the Ironomyiidae have been discovered in the Cenozoic (Fig. 1). The other families also have large gaps in their fossil records (Fig. 1), unlike the Phoridae, which are present in many deposits throughout the geological periods, with only two gaps in the Palaeocene and Pleistocene epochs (Fig. 1). However, this is to be expected as we have gaps in amber bioinclusions at these times [49].

The fact that wing variability is also a feature of sexual dimorphism in some living species, but not in the Cretaceous fossils, is another aspect to highlight. In some females, the wings are absent, atrophied or vestigial (extremely reduced) as in some *Chonocephalus* or *Cyphophorina* Borgmeier and Prado species, while males have well-developed wings [31]. However, to date, no fossil female phorid of the Cretaceous with a significant reduction of the wings has been reported. To investigate this further, we need to study additional Cretaceous specimens, focusing on finding males and females with similar morphologies preserved together in the same resin layer in single pieces.

The evolution of wings shows a significant modification in the morphology of Phoridae before (*early model*) and after (*recent model*) the K-Pg (Cretaceous–Paleogene) extinction event. This morphological change is reflected in the biodiversity of the major groups. The Sciadocerinae is a group that was highly diversified during the Cretaceous and had little biodiversity in the post-Cretaceous. This decline in biodiversity may be due to (1) changes in forest diversity that occurred during the Angiosperm Terrestrial Revolution (ATR, 100–50 Ma, [50]), which has impacted many groups of insects (e.g. [51]) such as ants (e.g. [52, 53]); (2) the survival of some populations in endemic places and the extinction of groups elsewhere; (3) a significant competition of territory or food resources between other groups of insects or other Phoridae. The absence of records for the group “†Prioriphorinae” Mostovski during the Cenozoic suggests a pair of alternative hypotheses: (1) the whole group disappeared during the ATR, and therefore the records are strictly Cretaceous, or (2) the group has been heavily impacted by its biodiversity as for Sciadocerinae, but their presence has not yet been discovered. The genus †*Agaphora* is strictly Cretaceous, but, as already mentioned, the wing shows characters of both models with radial veins (*early model*) and medial veins (*recent model*) that suggest it could be a “transitional” taxon and therefore belongs to the here-named “†Agaphorine-group.” The Euphorida were not present during the Cretaceous (except †*Metopina goeleti*) and had explosive radiation in the post-Cretaceous which suggests that the reduction of veins has favoured the explosion of the family. The evolution of the Phoridae favoured reduction, especially at the level of the wings with (1) shortening the radial veins and the proximal medial vein sector, and/or (2) the disappearance of the proximal medial vein sector leading to the disappearance of cells br, bm, and d, the cubital and anal sector veins (Additional file 1: Fig. S6). Moreover, some reductions observed on other structures such as femoral bristles are lost, or in males in abdominal segment 6 and terminalia, tergites are reduced and lost [25].

For the reduction of morphological structures in modern Phoridae, we propose two main hypotheses. The first is that a change in the environment with a predominance of Angiosperms after the ATR which has significantly altered the dynamics of the forests causing evolutionary pressures on insects [51]. The second is that co-evolution with other insect groups, e.g. with ants or termites, affected extinction-survival dynamics. It is well known that many Phoridae have a close relationship with ants or termites (e.g. [54–58]), thus the origin of complex sociality in ants and termites may have altered the food resources and reproduction opportunities for these flies (e.g. [59, 60]), potentially impacting their behaviour and morphology. Phorids that parasitise ants spend most of their time walking or flying short distances above the ground. Therefore, their wings do not require much strength or ventilation for flying. Phoridae that have changed to parasitic behaviour could result in a reduction in the number of veins. Hunting and predation behaviour during the Cretaceous that requires strength in the wings for a long flight would explain the need for a large number of veins to support its flights.

The interpretation of the phorid wing venation, and closely related families, has changed over time (Table 1), and accordingly, has an impact on the interpretation of the phylogenetic relationships (e.g. a re-described species can move into another genus as †*Maksika* Mostovski or can lead to the creation of a new genus as †*Hennigophora* Brown) [3, 4, 25, 31, 33, 61]. By redefining the wing vein terminology and creating two stable and unambiguous wing vein models, we propose a solution to this problem (Figs. 2 and 3). Our wing observations suggest that the Ironomyiidae are more similar to the Platypezidae than to the Phoridae (e.g. [39]), although Ironomyiidae is often considered to be an early family of Phoridae [25, 62–65]. The Opetiidae is more similar to the Lonchopteridae than to the other families, and the Lonchopteridae specimens have more plesiomorphic wing vein characters than other families.

The choice to develop two models is supported by the difference between the taxa mostly discovered in the Cretaceous (i.e. Sciadocerinae and “†Prioriphorinae”) and the rest of the Phoridae (i.e. clade Euphorida). The first model corresponds to phorids with a more complex wing vein pattern and little variability. Almost all Cretaceous species show this pattern, except for the genus †*Agaphora* and †*Metopina goeleti*. However, †*Agaphora* is probably a “transitional” genus between the Mesozoic and the Cenozoic, i.e. it possesses both characters identified as early and characters identified as recent, and †*Metopina goeleti* more probably comes from the Kinkora locality from the Magothy Formation, post-Cretaceous in age and containing modern fauna [63–65]. In contrast, few post-Cretaceous species show this pattern, namely only three species in Baltic amber and two living species, four of which belong to Sciadocerinae and †*Ulrichophora lobata*, sometimes included in the subfamily Sciadocerinae [2, 33]. Our results suggest the exclusion of †*Ulrichophora* from Sciadocerinae because of the lack of fusion of Sc to C, with the Sc running along RA until directly fused distally to C. This character is more similar to the Ironomyiidae with the Sc vein direction, except that it is not separated distally from RA before being fused to C. The majority of the Cretaceous species shows an early pattern, which is why we named this model the *early model*. The second model corresponds to the rest of the phorids, which show a venation pattern with important reductions. This model includes almost all post-Cretaceous species (i.e. Euphorida) with recent morphological characters (i.e. morphological characters found in the Cenozoic fossils and the living fauna), which gives the name *recent model*. In particular, many Baltic amber species in the genera *Hypocera* Lioy, *Gymnophora* Macquart, and *Megaselia* have these recent morphological characters. In contrast to the *early model*, the variability between species is very high, ranging from a slight reduction in all veins to the disappearance of all posterior veins (i.e. medial, post-cubital, and anal veins). For the few species that show a very significant vein reduction, we propose an example, as a support for labelling the veins, which should facilitate future research (Fig. 3c,d). In conclusion, the reduction of wing veins in Phoridae seems to occur first in the posterior part (i.e. anal veins then cubital veins) and then in the anterior part (i.e. medial vein then radial veins). Conversely, no tendency was observed for the distal part to have reduced veins before the proximal part, or vice versa.

We could not re-examine all the holotypes. Nevertheless, during the re-examination of certain holotypes, we noted errors of observation or interpretation of the wing veins, as for †*Prioriphora schroederhohenwarthi*, mainly due to the difficulty of observing the base of the wings, which requires strong luminosity or even more powerful tools (i.e. infrared microscopes or μ-CT scans). However, it will therefore be important, in future studies, to re-evaluate the fossil holotypes using new methods and applying new knowledge to verify the characters described, particularly the diagnostic characters to complement the descriptions and to have enough morphological characters for, for example, phylogenetic studies.

## Conclusions

The venation of insect wings has been studied and discussed with a foundation of constancy for 120 years, with revisions based on a variety of homology hypotheses. In recent years, new technologies have made it possible to develop new methods of observing things that were difficult to observe in the past, such as the base of the wings. Wing structures have evolutionary significance and are an important character pool for the study of new fossils. Our study provides a supporting tool for the description of morphological characters around the wings, and the clarification of the phorid wing venation, which may help the future descriptions of unknown fossil fauna as well as facilitate the identification of early fauna, in which the wing venation is a robust character for the identification. Thus, the homogenisation of wing vein terminology plays a pivotal role in advancing our understanding of the phylogenetic relationships of the Phoridae family, its relationships with outgroups, and the spatiotemporal evolution of the Phoridae as a whole. However, a future re-evaluation of the Cretaceous fauna is still necessary to clarify the diagnostic characters of each genus. In this respect, the numerous new phorids in amber from e.g. Myanmar and Spain will contribute to understanding this family’s early biodiversity.

## Methods

### Taxa examined

The three phorid holotypes re-evaluated in this study are: (1) †*Euliphora grimaldii* in Spanish amber in Álava locality (piece number is MCNA 8648, and information of piece in [66]), (2) †*Prioriphora schroederhohenwarthi* in Charentese amber in Archingeay-Les Nouillers locality (piece number is IGR-ARC-382.1b, and information of piece in [34]), and (3) †*Ulrichophora lobata* in Baltic amber (piece number is LACM ENT 159890, and information of piece in [2]). In Additional file 1, we show wing characters of fossil or living phorid specimens with different wing patterns, the figures are marked as “SX”, which have not yet been studied.

The isolate phorid wing found in Fouras-Bois Vert amber discovered by Dr Vincent Perrichot (University Rennes 1) is described here. This wing is preserved in the piece IGR-FRS-7, and it is housed in IGR. The Fouras-Bois Vert deposit is located in Charente-Maritime, western France, in the B2ms subunit belonging to the Upper Albian-Cenomanian [35, 67]. The locality Fouras-Bois Vert is also part of the set of ambers called “Charentese amber” [35, 68, 69]. The piece IGR-FRS-7 contain 53 arthropods and three conifer fragments as syninclusions (for a detailed list see [35]).

The first ironomyiid, in San Just amber (a wing), is described here. The wing is the first Ironomyiidae from the Albian period (113.0 to 100.5 Ma) and in Spanish amber. San Just is a locality of Urillas Municipality, in Teruel, Spain. The amber from San Just in northeastern Spain, within the Teruel Province, was first documented by Peñalver et al. [70] and was initially dated to the middle-upper Albian period [71]; however, recent comprehensive studies on palynomorphs suggest that it is more accurately placed in the upper Albian [72, 73]. The wing is preserved in the piece SJNB2012-12–10 (palaeontological excavation in 2012), housed in the Dinópolis collection (Teruel, Spain).

**Fig. 9:**
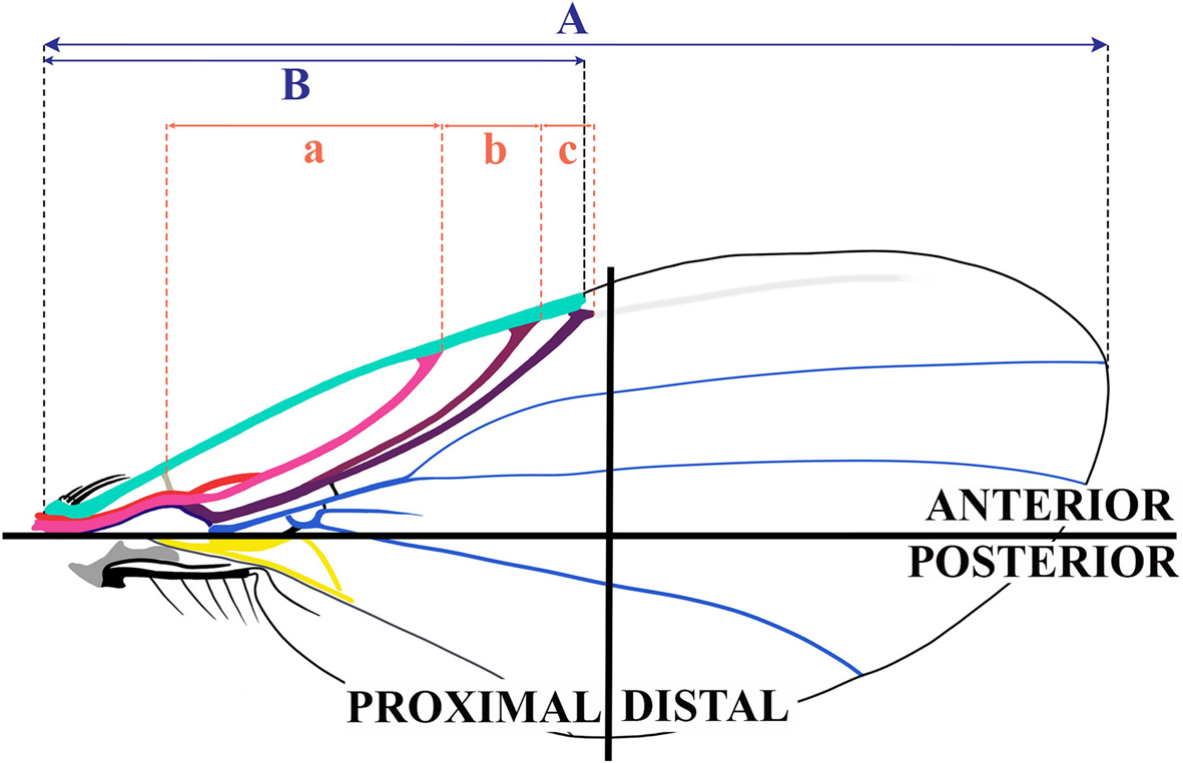
Wing orientation and vein distances. In black, the orientation of the wing. Blue line, length of costal (*B*) and wing (*A*) for calculating the CI. Orange line (*a*, *b*, *c*), the distance between crossvein h and radial veins for calculating the C_ratio_. The formula No. 1 with the CI and the formula No. 2 with the C_ratio_

Most of the amber pieces in this work are prepared and embedded in epoxy resin for preservation, following [74] and [75]. We use the wing orientation provided by the Cumming and Wood [13]: proximal to distal and anterior to posterior (Fig. 9). The Costal Index (CI) and Costal ratio (C_ratio_), represented in Fig. 9, are calculated by:

1 $$\text{CI}=\frac{\text{Costal length}\;(B)}{\text{Wing length}\;(A)}$$

2 $$\begin{aligned} {\mathrm C}_{\mathrm{ratio}} &=\frac{\mathrm{distance}\;\mathrm h-\mathrm{RA}\;\mathrm (a)}{\mathrm{distance}\;{\mathrm{RP}}_{1+2}-{\mathrm{RP}}_{3+4}\;\mathrm (c)}; \frac{\mathrm{distance}\;\mathrm{RA}-{\mathrm{RP}}_{1+2\;}\mathrm (b)}{\mathrm{distance}\;{\mathrm{RP}}_{1+2}-{\mathrm{RP}}_{3+4\;}\mathrm (c)};\;1 \end{aligned}$$

**Fig. 10:**
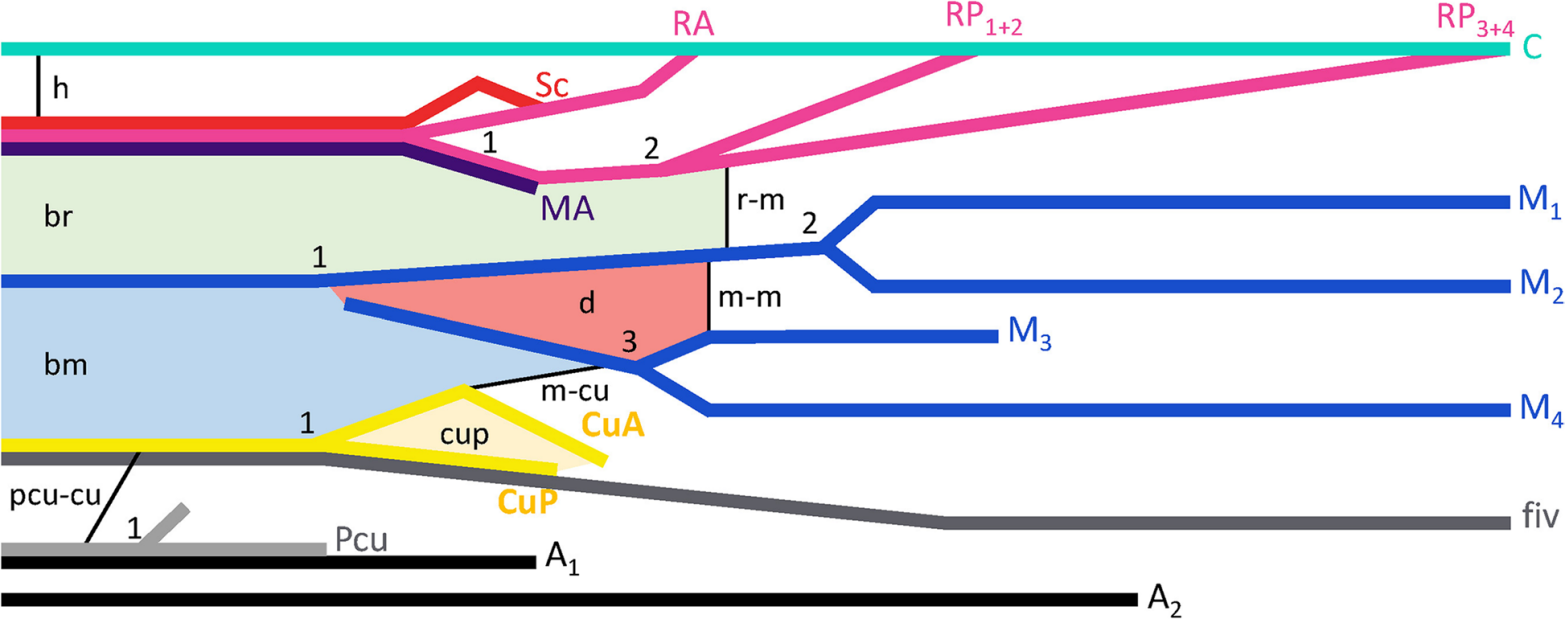
Schematic representation of forks and veins diagram of new combined terminology of Phoridae wings. Veins follow the colour code, the forks of radial, medial, cubital, and post-cubital veins are shown by the number. Crossveins are represented by a black trait. The cell by a coloured zone

The wing terminology of the two new models follows the schematic representation of forks and veins diagram in Fig. 10.

### Imaging and drawing

The photographs and Z-stacks images of the amber specimens were performed under a Nikon SMZ25 microscope, using Nikon SHR Plan Apo × 0.5 and SHR Plan Apo × 2 objectives with a microscope camera Nikon DS-Ri2 and the NIS-Element software (version 4.51.00 www.microscope.healthcare.nikon.com) at the SMF (Senckenberg Research Institute, Frankfurt am Main, Germany). Infrared-reflected photographs were taken with a Nikon Eclipse ME600D at the SMF (for precise technical information, see [76]). The living and fossil specimen pictures were photographed with a Nikon Z7II camera with a Nikon SHR Plan App WD:60 × 1 lens, both attached to the Nikon SMZ 25 stereomicroscope at the MNHN, and with a Keyence V-5000 imaging system at the Natural History Museum of Los Angeles County (LACM). The pictures were taken with the NControlPro 2 software and then processed on NX Studio; Helicon Focus software (version 7.6.1) was finally used to merge the pictures. Figures were performed using Adobe Photoshop software (version 25.4 www.adobe.com), and the Procreate software (version 5.3.7) on a tablet iPad Pro 10 (model A1876) was used for all digital drawings. The models were created from a combination of observations and do not correspond to any described species.

The original scan of †*Prioriphora schroederhohenwarthi* (see [34] for scan method) has been used for the re-evaluation of the wing, and it has been modified using VGStudioMax (version 3.3.1 www.volumegraphics.com/de, Volume Graphics, Heidelberg, Germany).

### Abbreviations (with colour codes)

Veins: *A*_1_: first anal vein (in black); *A*_2_: second anal vein (in black); *A*_3_: third anal vein (in black); *C*: costa vein (in cyan); *Cu*: cubitus vein; *CuA*: anterior branch of cubitus vein (in yellow); *CuA*_1_: anterior branch of first cubitus vein; *CuA*_2_: anterior branch of first cubitus vein; *CuA*+*CuP*: fusion of anterior branch and posterior branch of cubitus vein; *CuP*: posterior branch of cubitus vein (in shadow yellow); *fiv*: forewing intercalary vein (in shadow grey); *MA*: anterior branch of medial vein (in shadow blue); *MA*_1_: anterior branch of first medial vein; *MA*_2_: anterior branch of second medial vein; *M*_1_: first medial vein (in blue); *M*_2_: second medial vein (in blue); *M*_3_: third medial vein (in blue); *M*_4_: forth medial vein (in blue); *MP*: posterior branch of medial vein; *MP*_1_: posterior branch of first medial vein; *MP*_2_: posterior branch of second medial vein; *MP*_3_: posterior branch of third medial vein; *MP*_4_: posterior branch of fourth medial vein; *PCu*: postcubitus vein (in clear grey); *R*: radius vein; *R*_1_: first radius vein; *R*_2+3_: second and third radius vein; *R*_4+5_: fourth and fifth radius vein; *RA*: anterior branch of radius vein (in magenta); *RP*: posterior branch of radius vein (in purple); *RP*_1+2_: posterior branch of first and second radius vein (in purple); *RP*_3+4_: posterior branch of third and fourth radius vein (in purple); *Rs*: radius sector vein; *Rs*_1+2_: first and second radius sector vein; *Rs*_3+4_: third and fourth radius sector vein; *Sc*: subcostal vein (in red); *ScP*: posterior branch of subcostal vein.

Cells*:* *bm*: proximal medial cell; *br*: proximal radial cell; *cup*: posterior cubital cell; *d*: discal cell.

Crossveins: *h*: humeral crossvein (in beige); *cu-pcu*: cubital-postcubital crossvein (in black); *m-cu*: medial-cubital crossvein (in black); *m-m*: medial-medial crossvein (in black); *r-m*: radial-medial crossvein (in black).

## Supplementary Information

Additional file 1: Complementary Figures of Phoroidea wings, Figures S1–S7. Fig. S1 – Photographs of wings of living specimens with an *early model*. Fig. S2 – Photographs of wings of living specimens with a *recent model*. Fig. S3 – Wing of †*Agaphora iunior*. Fig. S4 – Photographs of wings of fossil specimens with an *early model*. Fig. S5 – Photographs of wings of fossil and extant specimens with a *recent model*. Fig. S6 – Hypothesis of reduction of Phoridae medial veins. Fig. S7 – Wing patterns of Lonchopteridae, Opetiidae, Platypezidae and Ironomyiidae.

Additional file 2. Additional of information on piece SJNB2012-12–10.

## Data Availability

All data generated or analysed during this study are included in this published article and its Additional files 1 and 2.
