# Supplementary material for: Review of wing morphology in fossil and modern species of humpbacked flies (Diptera: Phoridae)
Source: BMC Biol. 2025 Oct 7;23:298. doi: 10.1186/s12915-025-02376-8 (PMC12505603; doi:10.1186/s12915-025-02376-8)
Supplement: Supplementary file 1 — Additional file 1: Complementary Figures of Phoroidea wings, Figures S1–S7. Fig. S1 – Photographs of wings of living specimens with an early model. Fig. S2 – Photographs of wings of living specimens with a recent model. Fig. S3 – Wing of †Agaphora iunior. Fig. S4 – Photographs of wings of fossil specimens with an early model. Fig. S5 – Photographs of wings of fossil and extant specimens with a recent model. Fig. S6 – Hypothesis of reduction of Phoridae medial veins. Fig. S7 – Wing patterns of Lonchopteridae, Opetiidae, Platypezidae and Ironomyiidae. [file 12915_2025_2376_MOESM1_ESM.pdf]

Review of Wing Morphology in Fossil and Modern Species of Humpbacked Flies (Diptera: Phoridae)

Mélanie C.M. Herbert[1*], André Nel[2], Brian V. Brown[3], Antonio Arillo[4], Brendon E. Boudinot[5], Mónica M. Solórzano-Kraemer[1]

[1]*Paläontologie und Historische Geologie, Senckenberg Forschunginstitut und Naturmuseum, Senckenberganlage 25, D-60325 Frankfurt-am-Main, Germany*.

[2]*Institut Systématique Evolution Biodiversité (ISYEB), Muséum national d'Histoire naturelle, CNRS, Sorbonne Université, EPHE, Université des Antilles, Paris, France.*

[3]*Department of Entomology, Natural History Museum of Los Angeles County, 900 Exposition Blvd, Los Angeles, CA, 90007, USA.*

[4]*Departamento de Biodiversidad, Ecología y Evolucíon, Facultad de Biología, Universidad Complutense, Madrid, Spain.*

[5]*Entomology II, Abteilung Terrestrische Zoologie, Senckenberg Forschungsinstitut und Naturmuseum, Senckenberganlage 25, D-60325 Frankfurt-am-Main, Germany.*

[*]*Correspondence to be sent to: Paläontologie und Historische Geologie, Senckenberg Forschunginstitut und Naturmuseum, Senckenberganlage 25, D-60325 Frankfurt-am-Main, Germany. E-mail address:* melanie.herbert@senckenberg.de

ADDITIONAL FILE 1

Complementary Figures of Phoroidea wings

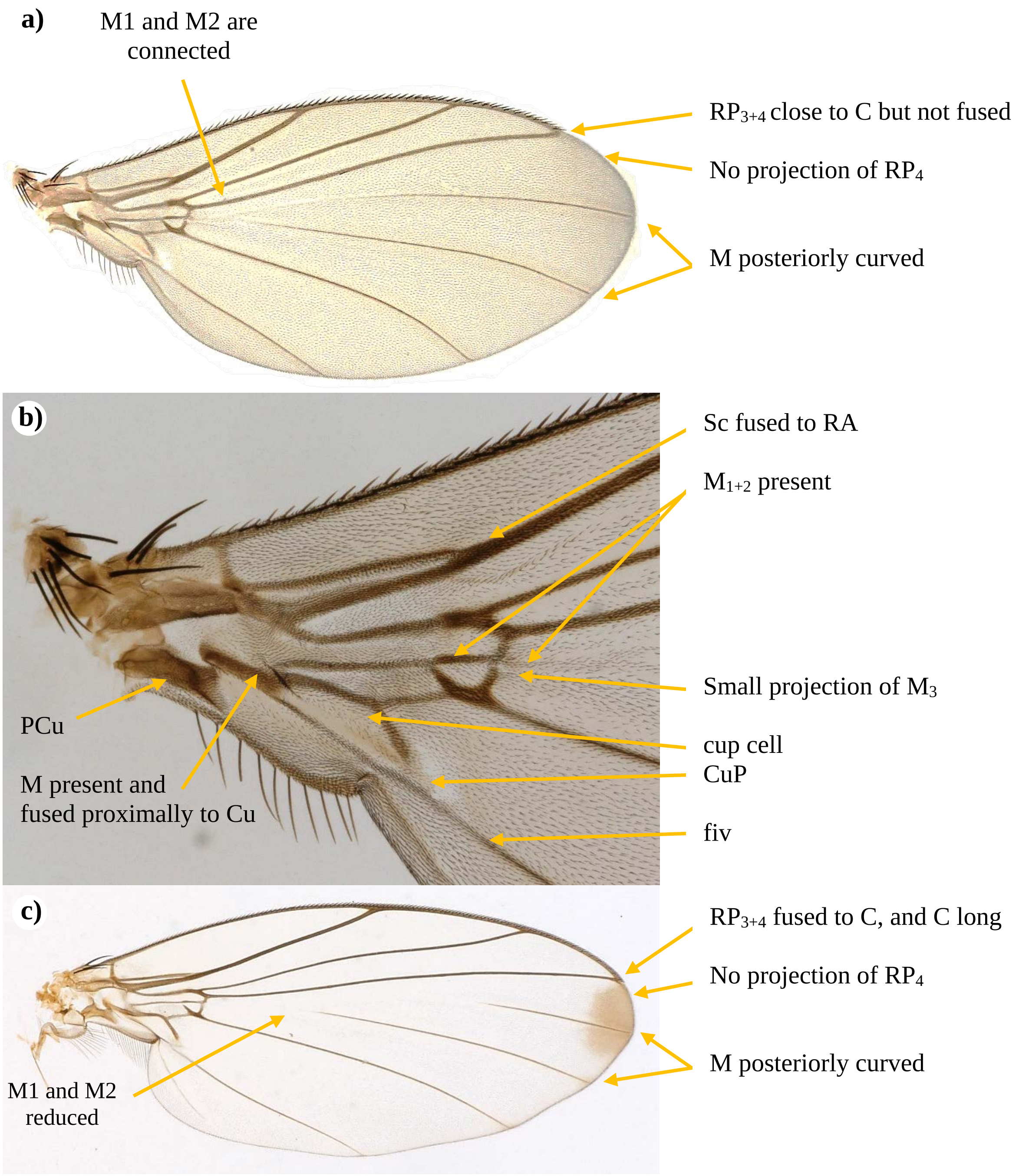

Figure S1. **Photographs of wings of living specimens with an early model**. *Archisciada* sp., LACM no number: a, full wing; b, zoom of basal part; c, *Sciadocera* sp., male, LACM no number

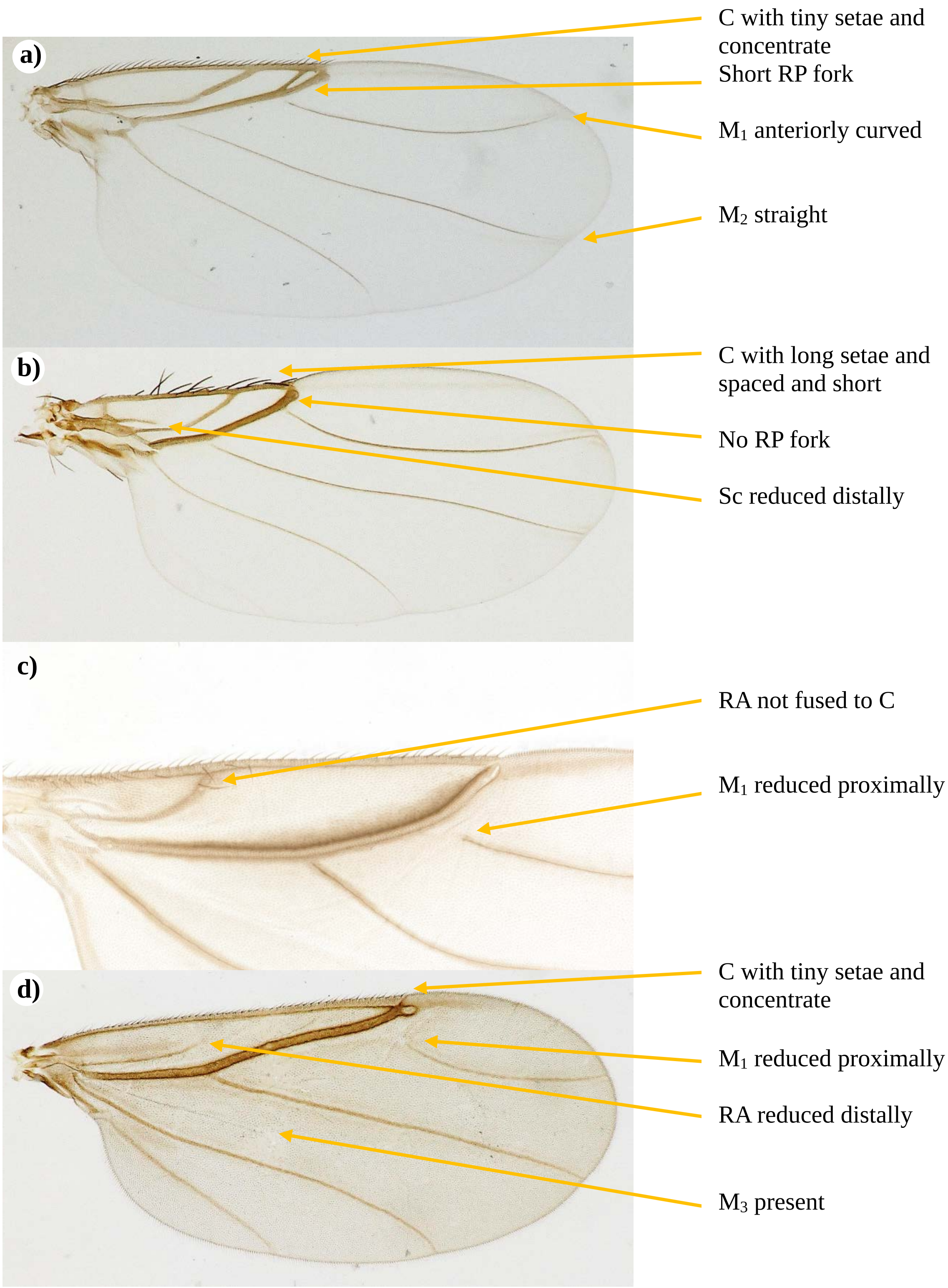

Figure S2. **Photographs of wings of Living specimens with a recent model**. a, *Dohrniphora* sp., LACM No number; b, *Conicera* sp., LACM No number; c, *Metopina* sp., LACM No number; d, *Chronocephalus* sp., LACM No number.

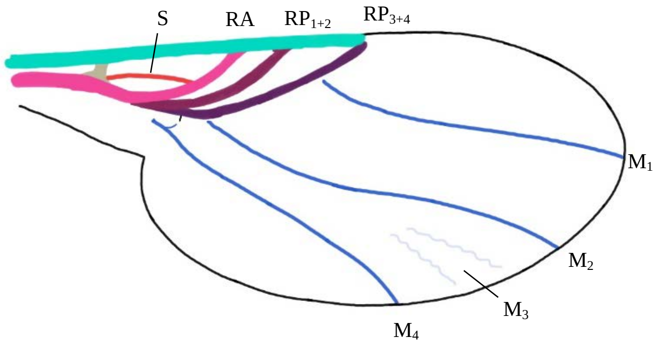

Figure S3. Wing of †***Agaphora iunior*** **Mostovski, 1999**. Drawing based on figure 7 in Mostovski (1999)

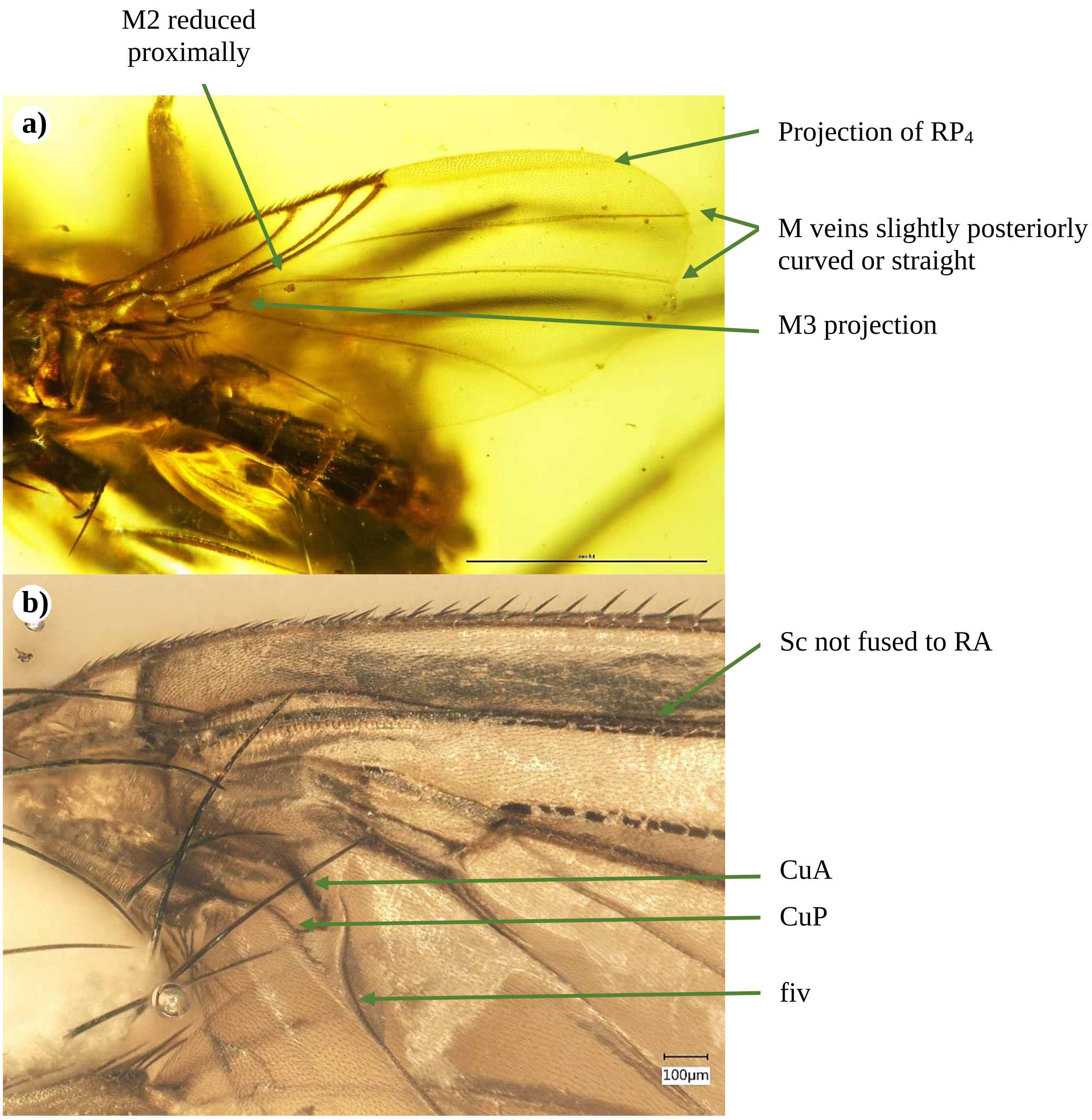

Figure S4. **Photographs of wings of fossil specimens with an early model**. a, †*Prioriphora* sp. SMF-Be 2381.2 in Burmese amber; b, potentiel †*Ulrichophora* sp. CCHH 4804-3 (LACM) in Baltic amber.

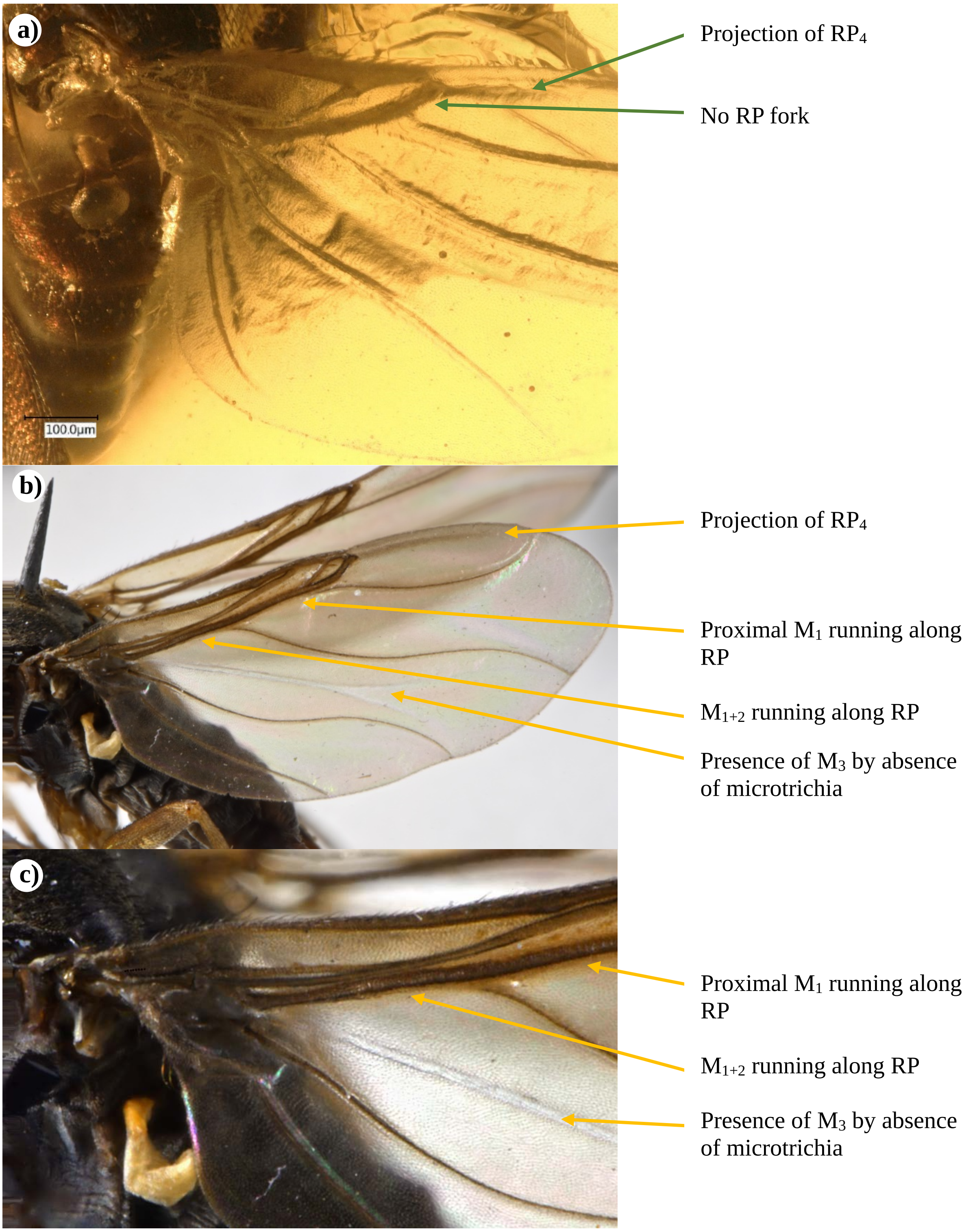

Figure S5. **Photographs of wings of fossil and extant specimens with a recent model**. a, Potentiel †*Aenigmatias* sp. CCHH 1804-1 (LACM), in Baltic amber; b and c, *Gymnophora* sp., MNHN no number, living species, full wing and zoom of proximal part, respectfully.

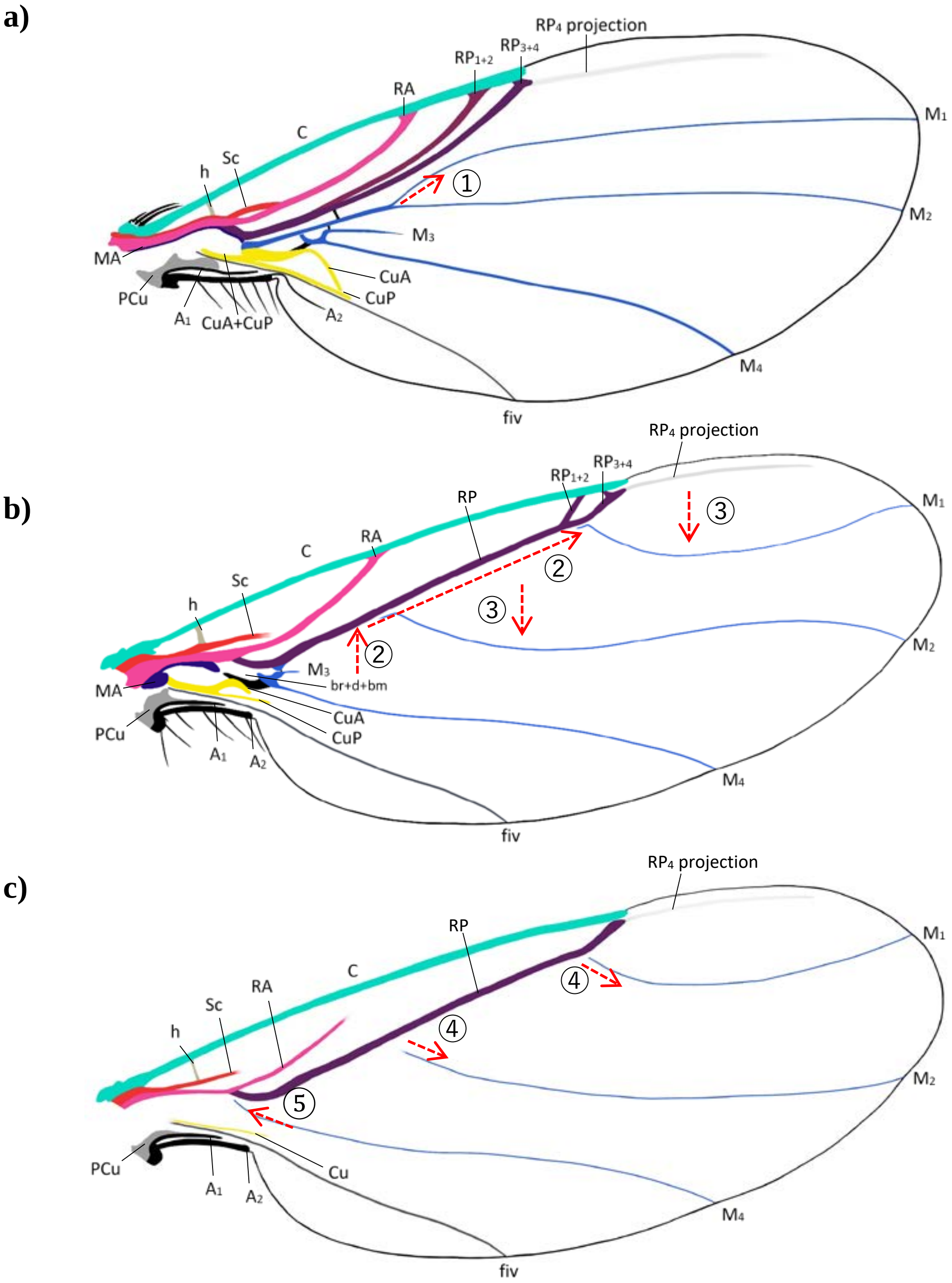

Figure S6. **Hypothesis of reduction of Phoridae medial veins**. a, Early wing, M_1 and M_2 are clearly separate from RP, and M_1 is connected to M_2 or reduced proximally ①, and all three proximal cells are present; b, Recent wing, RP fork reduced ②, M_1 and M_2 migrated to the posterior radial system without fused to RP veins ②. Opening of the proximal cells and a change in their orientation from posteriorly curved to sigmoidal ③; c, Wing with an "extreme"

reduction of venation, proximal reduction of M_1 and M_2 ④, M_4 extending proximally ⑤, disappearance of the cells.

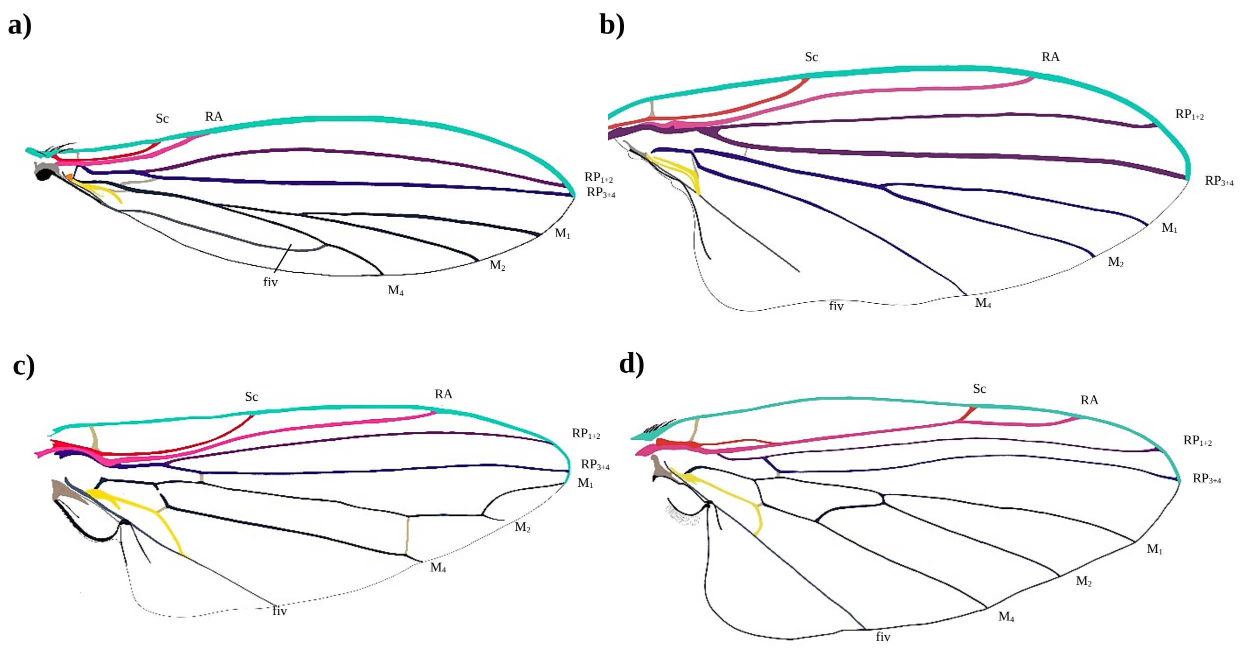

Figure S7. **Wing patterns**. a) Lonchopteridae, b) Opetiidae, c) Platypezidae, d) Ironomyiidae.
