# Supplementary material for: Review of wing morphology in fossil and modern species of humpbacked flies (Diptera: Phoridae)
Source: BMC Biol. 2025 Oct 7;23:298. doi: 10.1186/s12915-025-02376-8 (PMC12505603; doi:10.1186/s12915-025-02376-8)
Supplement: Supplementary file 2 — Additional file 2. Additional of information on piece SJNB2012-12–10. [file 12915_2025_2376_MOESM2_ESM.pdf]

Review of Wing Morphology in Fossil and Modern Species of Humpbacked Flies (Diptera: Phoridae)

Mélanie C.M. Herbert[1*], André Nel[2], Brian V. Brown[3], Antonio Arillo[4], Brendon E. Boudinot[5], Mónica M. Solórzano-Kraemer[1]

[1]*Paläontologie und Historische Geologie, Senckenberg Forschunginstitut und Naturmuseum, Senckenberganlage 25, D-60325 Frankfurt-am-Main, Germany.*

[2]*Institut Systématique Evolution Biodiversité (ISYEB), Muséum national d'Histoire naturelle, CNRS, Sorbonne Université, EPHE, Université des Antilles, Paris, France.*

[3]*Department of Entomology, Natural History Museum of Los Angeles County, 900 Exposition Blvd, Los Angeles, CA, 90007, USA.*

[4]*Departamento de Biodiversidad, Ecología y Evolucíon, Facultad de Biología, Universidad Complutense, Madrid, Spain.*

[5]*Entomology II, Abteilung Terrestrische Zoologie, Senckenberg Forschungsinstitut und Naturmuseum, Senckenberganlage 25, D-60325 Frankfurt-am-Main, Germany.*

[*]Correspondence to be sent to: Paläontologie und Historische Geologie, Senckenberg Forschunginstitut und Naturmuseum, Senckenberganlage 25, D-60325 Frankfurt-am-Main, Germany. E-mail address: melanie.herbert@senckenberg.de

ADDITIONAL FILE 2

Additional of information on piece SJNB2012-12-10

Specimen SJNB2012-12-10 comes from a large piece that has been divided into small fragments. Each fragment has a letter and each bioinclusion has a catalogue number. Pieces with more than one inclusion are surrounded by a square.

Fragments 12-25, 12-26, 12-27, 12-28, 12-30, 12-31, 12-32, 12-34, 12-35 and 12-36 were used to describe the species *Protoculicoides hispanicus* and *Protoculicoides sanjusti* (Diptera Ceratopogonidae). The reference publication is: Szadziewski, R., Arillo, A., Urbanek, A., & Sontag, E. (2016) Biting midges of the extinct genus *Protoculicoides* Boesel from Lower Cretaceous amber of San Just, Spain and new synonymy in recently described fossil genera (Diptera: Ceratopogonidae). *Cretaceous Research*, 100(58), 1–9. https://doi.org/10.1016/j.cretres.2015.09.016

<u>Piece SJNB2012-12:</u>

SJNB2012 12-01 Homoptera	(v)
SJNB2012 12-02 Psocoptera	(R)
SJNB2012 12-03 Psocoptera	(S)
SJNB2012 12-04 Hymenoptera Scelionidae	(j)
SJNB2012 12-05 Hymenoptera Scelionidae	(k)
SJNB2012 12-06 Psocoptera	(T)
SJNB2012 12-07 Araneae Lagonomegopidae	(z1)
SJNB2012 12-08 Hymenoptera Mymarommatoidea	
SJNB2012 12-09 Diptera Chironomidae	(o)
SJNB2012 12-10 Diptera Ironomyiidae (wing)	(q)
SJNB2012 12-11 Diptera Ceratopogonidae	(p)
SJNB2012 12-12 Araneae	(n)
SJNB2012 12-13 Hymenoptera Scelionidae	(L)
SJNB2012 12-14 Homoptera	(u)
SJNB2012 12-15 Hymenoptera Scelionidae	(m)
SJNB2012 12-16 Insecta indet.	(x)
SJNB2012 12-17 Thysanoptera	(F)
SJNB2012 12-18 Homoptera exuvia?	(w)
SJNB2012 12-19 Araneae	(z2)
SJNB2012 12-20 Hymenoptera Scelionidae	(z3)

SJNB2012 12-21 Hymenoptera Scelionidae (z4)
SJNB2012 12-22 Hymenoptera Scelionidae (z5)
SJNB2012 12-23 Hymenoptera Scelionidae (z6)
SJNB2012 12-24 Diptera Rhagionidae (i)
Litoleptis fossilis
SJNB2012 12-25 Diptera Ceratopogonidae
Protoculicoides hispanicus ♂
SJNB2012 12-26 Diptera Ceratopogonidae
Protoculicoides hispanicus ♂
SJNB2012 12-27 Diptera Ceratopogonidae (e)
Protoculicoides hispanicus ♀
SJNB2012 12-28 Diptera Ceratopogonidae (g)
Protoculicoides hispanicus ♀
SJNB2012 12-29 Spider web (or fungus) (y)
SJNB2012 12-30 Diptera Ceratopogonidae (h)
Protoculicoides hispanicus ♀
SJNB2012 12-31 Diptera Ceratopogonidae (a)
Protoculicoides sanjusti ♂HOLOTYPE
SJNB2012 12-32 Diptera Ceratopogonidae (c)
Protoculicoides hispanicus ♂HOLOTYPE
SJNB2012 12-33 Diptera
SJNB2012 12-34 Diptera Ceratopogonidae (b)
Protoculicoides hispanicus ♂PARATYPE
SJNB2012 12-35 Diptera Ceratopogonidae
Protoculicoides hispanicus ♀ PARATYPE
SJNB2012 12-36 Diptera Ceratopogonidae (d)
Protoculicoides sanjusti ♂PARATYPE
